# Targeted Lipidomics of Mitochondria in a Cellular Alzheimer’s Disease Model

**DOI:** 10.3390/biomedicines9081062

**Published:** 2021-08-21

**Authors:** Irina Kurokin, Anna Andrea Lauer, Daniel Janitschke, Jakob Winkler, Elena Leoni Theiss, Lea Victoria Griebsch, Sabrina Melanie Pilz, Veronika Matschke, Martin van der Laan, Heike Sabine Grimm, Tobias Hartmann, Marcus Otto Walter Grimm

**Affiliations:** 1 Experimental Neurology, Saarland University, 66421 Homburg, Germany; irina.k.10e@gmx.de (I.K.); anna.lauer@uks.eu (A.A.L.); daniel.janitschke@uks.eu (D.J.); jakob.winkler@uks.eu (J.W.); elena.theiss@web.de (E.L.T.); lea@ifuws.de (L.V.G.); pilz.sabrina1995@gmail.com (S.M.P.); heike.grimm@gmx.de (H.S.G.); 2 Department of Cytology, Institute of Anatomy, Medical Faculty, Ruhr University Bochum, D-44801 Bochum, Germany; Veronika.Matschke@rub.de; 3 Medical Biochemistry & Molecular Biology, Center for Molecular Signaling PZMS, Saarland University Medical School, 66421 Homburg, Germany; martin.van-der-laan@uks.eu; 4 Deutsches Institut für Demenzprävention, Saarland University, 66421 Homburg, Germany; tobias.hartmann@uks.eu; 5 Nutrition Therapy and Counseling, Campus Rheinland, SRH University of Applied Health Sciences, 51377 Leverkusen, Germany

**Keywords:** mitochondria, Alzheimer’s disease, neurodegeneration, lipidomics, unsaturated fatty acids, phosphatidylcholine, phosphatidylethanolamine, lyso-phospholipids, plasmalogens, carnitine carrier system

## Abstract

Alzheimer’s disease (AD) is neuropathologically characterized by the accumulation of Amyloid-β (Aβ) in senile plaques derived from amyloidogenic processing of a precursor protein (APP). Recently, changes in mitochondrial function have become in the focus of the disease. Whereas a link between AD and lipid-homeostasis exists, little is known about potential alterations in the lipid composition of mitochondria. Here, we investigate potential changes in the main mitochondrial phospholipid classes phosphatidylcholine, phosphatidylethanolamine and the corresponding plasmalogens and lyso-phospholipids of a cellular AD-model (SH-SY5Y APPswedish transfected cells), comparing these results with changes in cell-homogenates. Targeted shotgun-lipidomics revealed lipid alterations to be specific for mitochondria and cannot be predicted from total cell analysis. In particular, lipids containing three and four times unsaturated fatty acids (FA X:4), such as arachidonic-acid, are increased, whereas FA X:6 or X:5, such as eicosapentaenoic acid (EPA) or docosahexaenoic acid (DHA), are decreased. Additionally, PE plasmalogens are increased in contrast to homogenates. Results were confirmed in another cellular AD model, having a lower affinity to amyloidogenic APP processing. Besides several similarities, differences in particular in PE species exist, demonstrating that differences in APP processing might lead to specific changes in lipid homeostasis in mitochondria. Importantly, the observed lipid alterations are accompanied by changes in the carnitine carrier system, also suggesting an altered mitochondrial functionality.

## 1. Introduction

Alzheimer’s disease (AD) is a devastating neurodegenerative disorder, histopathologically characterized by extracellular amyloid plaques and intraneuronal neurofibrillary tangles in brain regions such as hippocampus and cortex [1,2]. Amyloid plaques are mainly composed of aggregated Amyloid-β (Aβ) peptides released by sequential proteolytic processing of the amyloid precursor protein (APP) by β- and γ-secretase [3,4]. However, increasing evidence suggests that small oligomers of Aβ up to 50 Aβ subunits represent the most toxic species of Aβ [5,6,7]. Several mechanisms of Aβ toxicity finally resulting in reduced neuronal function and viability, synaptic degeneration, cell death and memory loss are discussed, including, e.g., disruption of calcium homeostasis, changes in lipid homeostasis, inflammation as well as increased oxidative stress and mitochondrial dysfunction [8,9,10,11]. In line with the finding that energy metabolism is impaired in AD and precedes the clinical onset of AD, mitochondrial dysfunction is discussed to be an early and major aspect of the disease [12,13]. Mitochondrial abnormalities observed in AD include impaired mitochondrial bioenergetic, increased oxidative stress and disturbed mitochondrial genome. However, it is controversially discussed whether Aβ induces AD mitochondrial dysfunction or whether mitochondrial dysfunction is independent of Aβ [12]. Based on the multifactorial aspect of AD, it is more likely that pleiotropic mechanisms interfere with each other or exist side by side, finally leading to pathological changes. For example, Aβ has been shown in cell culture and animal models to enhance cytoplasmic calcium levels, and elevated calcium, in turn, interferes with mitochondrial function, reducing ATP production [14,15,16,17]. Decreased energy or ATP level are a common characteristic of AD [18,19]. Aβ itself contains a mitochondrial targeting motif [20,21], is located within mitochondria [22] and impairs respiratory chain function in isolated mitochondria [23,24,25]. The impact of APP and its processing products on mitochondrial function is further substantiated by the finding that the APP intracellular domain (AICD) upregulates the expression of the mitochondrial master transcriptional coactivator PGC-1α and that impairments in this pathway may lead to mitochondrial dysfunction and neurodegeneration [26]. Beside the expression of PGC1α, AICD has been reported to regulate the expression of several genes, including genes involved in the regulation of lipid homeostasis, and changes in lipid homeostasis are observed in AD [27,28,29,30,31,32,33].

As mentioned above, several lines of evidence suggest a link between mitochondrial function and AD on the one hand and lipid homeostasis and AD on the other hand. However, very few data exist concerning whether the lipid composition in mitochondria is also altered in AD and if this might be associated with mitochondrial function such as the carnitine carrier system. In addition, nothing is known if potential alterations in the lipid composition are specific for mitochondria or if mitochondrial changes in lipid homeostasis just reflect known lipid alterations of the complete tissue or cell homogenate.

As AD cellular model we used neuroblastoma cells overexpressing the familial APPswedish mutation, known to be primarily processed by β-secretase, thus leading to elevated Aβ level and transcriptional active AICD [29,34,35,36]. Interestingly, regulation of gene transcription of AICD in lipid homeostasis has been reported, but other cellular processes are also highly affected by AICD. Importantly, differences in gene regulation of AICD derived from amyloidogenic or non-amyloidogenic processing have been observed [29,36]. Therefore, results obtained by APPswedish overexpressing cells were compared to a second cell line overexpressing APPwt, which is mainly processed by the non-amyloidogenic α-secretase pathway to address the potential implications of differences in APP processing on mitochondrial lipid homeostasis.

In this semi-quantitative targeted lipidomics approach, phospholipids including phosphatidylcholine (PCaa), phosphatidylethanolamine (PEaa), plasmalogens (PCae, PEae), lyso-phospholipids (lyso-PC, lyso-PE) and components of the carnitine carrier system were analyzed. PCaa and PEaa are lipid species, which represent approximately 80% of the phospholipid species present in mitochondria [37]. Plasmalogens have been found to be affected in AD [38,39,40,41,42]. Furthermore, AICD has been shown to regulate the expression of the alkyl-dihydroxyacetonephosphate-synthase, a rate limiting enzyme in plasmalogen synthesis, and plasmalogens interfere with APP processing by inhibiting γ-secretase activity [33,43]. Lyso-PC species were also found to be altered in AD. Notably, in AD brain, lyso-PC species have been found to be decreased [40,44,45,46], whereas phospholipase A2 activity highly expressed in different brain regions has been reported to be elevated in AD [47,48,49,50]. As mentioned above, potential changes in the lipid composition might also affect the energy metabolism and mitochondrial function. Therefore, we extended our study to acetyl- and acyl-carnitine. Fatty acids are transported as acyl-carnitines via the carnitine carrier system in mitochondria to provide them to β-oxidation. As a final product of β-oxidation, acetyl-carnitine is shuttled back to cytosol. Hence, alterations in acyl-carnitine or acetyl-carnitine or the corresponding ratios indicate a change in β-oxidation or the carnitine carrier system.

## 2. Materials and Methods

### 2.1. Chemicals and Reagents

HPLC-grade pyridine, phenyl isothiocyanate (PITC) and ammonium acetate used for lipid extraction were purchased from Merck (Darmstadt, Germany). If not stated otherwise, all other chemicals were purchased from Fisher Scientific (Schwerte, Germany).

### 2.2. Cell Culture

SH-SY5Y cells transfected with the pCEP4 expression vector alone (mock), pCEP4-APPwt or pCEP4-APPswedish, respectively, were cultivated in humified incubator at 37 °C and 5% CO_2_ in Dulbecco’s Modified Eagle’s Medium (DMEM) containing 10% FBS (GE Healthcare Life Sciences, Chalfont St. Giles, UK), 0.1 mM non-essential amino acids and Hygromycin B (400 µg/mL).

### 2.3. Isolation of Mitochondrial Fraction

Cells grown on a 100 cm^2^ flask were cultivated as described above until 100% confluence before the isolation of the mitochondrial fraction was performed at 4 °C. Cells were washed twice with ice-cold 1x PBS and harvested in 500 µL 1× PBS followed by centrifugation at 800× *g* for 5 min. The cell pellet was washed using 500 µL 1× PBS and afterwards resuspended in 1 mL buffer A (83 mM sucrose, 10 mM HEPES, pH 7.2). Cells were lysed mechanically via Minilys (Peqlab, Erlangen, Germany) using ceramic beads for 5 s at low power firstly, and after adding 1 mL of Buffer A, they were homogenized using 30 strokes of Potter at maximal rounds per minute. Before differential centrifugation at 1000× *g* for 5 min was performed, 2 mL buffer B (25 mM sucrose, 30 mM HEPES, pH 7.2) were added to the homogenates. Supernatant was collected and centrifuged at 1300× *g* for 5 min and pellet discarded. The mitochondrial fraction was isolated by centrifugation of the supernatant at 12,000× *g* for 2 min. Mitochondrial pellet was resuspended in 1 mL buffer B and differential centrifugation was repeated as described with the last centrifugation step at 21,000× *g* for 20 min. The mitochondrial pellet was then resuspended in 100 µL buffer C (320 mM sucrose, 1 mM EDTA, 10 mM Tris-Cl, pH 7.4) and the protein concentration was determined using the Bicinchoninic acid assay according to Smith et al. [51]. Then, 1 U proteinase K per 1 mg/mL protein was added to the mitochondria and samples were incubated for 45 min in a 37 °C water bath. Proteinase K treatment was stopped adding 10 µL of the protease inhibitor PMSF (200 mM in 2-propanol, 37 °C) and incubated for 20 min on ice. Afterwards, differential centrifugation was performed as described before (1000× *g* for 5 min; 1300× *g* for 5 min; 21,000× *g* for 20 min) and the mitochondria pellet resuspended in 100 µL buffer C.

### 2.4. Western Blot Analysis

To confirm the isolation of purified mitochondria and the overexpression of APP in the used cells, mitochondria and cell lysates were prepared as described above. Mitochondria pellets and lysates were adjusted to equal protein amounts by determining the protein concentrations in samples with the bicinchoninic acid assay according to Smith et al. [51]. Samples were loaded on 10–20% tris-tricine-gradient gels (Anamed Elektrophorese, Groß-Bieberau, Germany) and thereafter, proteins were transferred onto nitrocellulose membranes (Whatman, Dassel, Germany). The used antibodies for the subsequent analysis are listed in Table 1 and the enhanced chemiluminescence (ECL-) method (Perkin Elmer, Rodgau-Jügesheim, Germany) was used for signal detection. Densitometrical quantification of band intensity after subtraction of the background signal was performed with the Image Gauge 3.45 software (Fujifilm, Düsseldorf, Germany).

### 2.5. Verification of the Mitochondrial Sample by Transmission Electron Microscopy

TEM studies were performed to validate sample purity of mitochondrial isolation out of SH-SY5Y cells. To examine the mitochondrial structure in transfected cells, cells were plated in T25 cell culture flasks and cultivated as described above until 100% confluence. Isolated mitochondria as well as cells were fixated with 2% formaldehyde (#15714-S, Electron Microscopy Sciences, Hatfield, PA, USA), 2.5% glutaraldehyde (#G5882, Merck, Darmstadt, Germany) and 2mM CaCl_2_ in 0.15 mM cacodylate buffer. Subsequently samples were stained with 1% osmium tetroxide in H_2_O for 1 h. Thereafter, cells were detached from the flasks bottom with cell scrapers and centrifuged at 1800 rpm for 3 min. Afterwards, cell bands were embedded in Agar-Agar (3% in PB). Next, specimens were dehydrated through an ascending ethanol series, starting with 50% ethanol, followed by incubation in 70% ethanol, 1% uranyl acetate (#21447, Polyscience Europe, Heidelberg, Germany) and 1% phosphotungstic acid (#455970, Merck, Darmstadt, Germany) solution over night at 4 °C. The next day, dehydration continued with an ascending ethanol series (80–100%). The specimens were carefully transferred into epoxy resin. This was accomplished by first incubating the tissue in propylene oxide (#807027, Merck, Germany), followed by an ascending series of propylene oxide and EPON mixtures. This embedding procedure started with propylene oxide/EPON in a 3:1 ratio, followed by a 1:1 ratio, and ended with a 1:3 ratio. Finally, specimens were penetrated by pure EPON over night at 20 °C. On the third day of embedding, EPON was renewed. The EPON embedded specimens were allowed to polymerize at 60 °C for two days. EPON consists of glycidether (#21045.02, Serva, Heidelberg, Germany), methylnadic anhydride (#29452.02, Serva, Heidelberg, Germany), 2-dodecenylsuccinic acid anhydride (#20755.01, Serva, Heidelberg, Germany) and 2,4,6-Tris(dimethylaminomethyl)phenol (#36975.01, Serva, Heidelberg, Germany) in a 5,4:3,8:1,84:1 mixture. Ultrathin slices (70 nm) were cut with an Ultracut E Reichert-Jung (Leica Microsystems GmbH, Wetzlar, Germany) with a DiATOME histo diamond knife (45°, 6 mm, MX559; Diatome AG, Nidau, Switzerland). Images were recorded on a JEOL JEM-1400 Plus transmission electron microscope (JEOL, Akishima (Tokyo), Japan) operating at a 120 kV with a LaB6 filament and equipped with a 4096 × 4096-pixel CMOS camera (TemCam-F416, TVIPS, Gauting, Germany).

### 2.6. Lipid Extraction and Mass Spectrometry

Lipids were analyzed by a shotgun mass spectrometry approach which means that after lipid extraction, lipids were analyzed by a specific SRM (selected reaction monitoring) method. For each lipid, a specific fragment and the according precursor ion were detected (see Supplementary Table S1). Immediately before the lipids were extracted using the solid/liquid extraction method described in detail earlier [40,52,53], the mitochondria membranes were disrupted mechanically via Minilys (Peqlab, Erlangen, Germany). In brief, a mixture of the internal standards (see Table 2) followed by the mitochondrial samples were added to circles of Whatman blotting paper with a diameter of 6 mm, that were placed on the wells of a 96-well filter plate (0.45 µm; Merck, Darmstadt, Germany) fixed on a 96-deep well plate (Fisher Scientific, Schwerte, Germany). After drying the samples under a nitrogen flow (1–2 bar) for 45 min, 20 µL of 5% phenyl isothiocyanate (*v/v*) diluted in ethanol/water/pyridine (1:1:1, *v/v/v*) were added and the samples were incubated for 20 min at room temperature before they were dried as described before. The extraction of lipids was performed using 300 µL 4.93 mM ammonium acetate in methanol and shaking the plate for 30 min at 450 rpm on a plate shaker (IKA, Staufen, Germany). The lipids were transferred into the 96-deep well plate by centrifugation for 2 min at 500× *g* and the samples were diluted with 600 µL 5 mM ammonium acetate in methanol/water (97:3, *v/v*) in the 96-deep well plate. After covering the plate with a silicone mat, it was shaken for 2 min at 450 rpm at room temperature before targeted shotgun mass spectrometry analysis was performed.

A 4000-quadropole linear-ion trap (QTrap) equipped with a turbo spray ion source (AB Sciex, Darmstadt, Germany) was used for the detection of different lipid species, including phospholipids and carnitines. Measurements were performed in triplicates using the Analyst 1.4.2 software (AB Sciex, Darmstadt, Germany) with the help of an autosampler of the Agilent HPLC 1200 series as described in detail in [40,52,53]. Q1/Q3 masses, declustering potentials and collision energies for the analyzed metabolites are listed in Supplementary Table S1. Potential matrix effects were calculated by building the ratio between the deuterated lipid standards in presence of lipid extracts from mock, SH-SY5Y APPwt- and SH-SY5Y APPswedish cells. The change in the ratio was maximum 2.97% for homogenates and 2.39% for mitochondria and in average 1.19% (see Supplemental Figure S2A,B).

### 2.7. Detection of Cardiolipin

The Cardiolipin Assay Kit from abcam (ab241036; Berlin, Germany) was used to analyze the levels of cardiolipin in SH-SY5Y mock and APPswedish transfected cells according to the manufacturer’s instructions.

### 2.8. Statistical Analysis

The Analyst 1.4.2 software from AB Sciex (Darmstadt, Germany) was used to extract the counts per second for each MRM pair and each lipid was normalized to its respective lipid class standard. The mean per technical triplicate was formed for each lipid/standard ratio per sample (*n* = 7, respectively). To analyze the distribution of a lipid species within a lipid class, the lipid species signals were divided by the sum of the lipid class of the lipid species. R (R Core Team 2020; Vienna, Austria; https://www.R-project.org/; accessed on 4 May 2021) was applied to perform statistical analysis. Two-tailed Student’s *t*-test was used for *p* value calculation for each parameter, shown in volcano plots, which were created via the R package “EnhancedVolcano” (Kevin Blighe, Sharmila Rana and Myles Lewis (2020) version 1.6.0. https://github.com/kevinblighe/EnhancedVolcano; accessed on 4 May 2021). Fisher’s exact test was used to determine if the increased/decreased lipid distribution significantly differs between mitochondria and homogenate. Error bars graphs represent standard error of the mean. Significance was set at * *p* ≤ 0.05, ** *p* ≤ 0.01 and *** *p* ≤ 0.001.

## 3. Results

### 3.1. Lipid Analysis in Isolated Mitochondria Fraction and Total Cell Homogenate of APPswedish Transfected Human Neuroblastoma Cells

**Figure 1:**
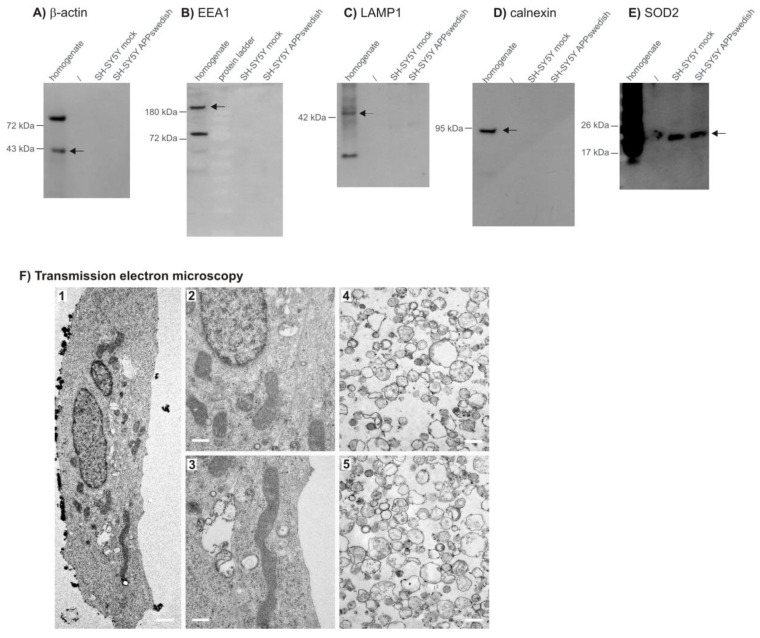
Verification of the mitochondrial preparations by Western blot analysis and transmission electron microscopy. (**A**) Protein level of β-actin in homogenate (lane 1) and the absence of this major component of the cytoskeleton in samples of isolated mitochondria from SH-SY5Y mock- or APPswedish transfected cells (lanes 3–4). (**B**) Early Endosomal Antigen 1 (EEA1) protein level in homogenate (lane 1) and the absence of this early endosome-associated protein in samples of isolated mitochondria from SH-SY5Y mock or APPswedish transfected cells (lanes 3–4). (**C**) Protein level of LAMP1 in homogenate (lane 1) and the absence of this lysosomal-associated membrane protein in samples of isolated mitochondria from SH-SY5Y mock or APPswedish transfected cells (lanes 3–4). (**D**) Calnexin protein level in homogenate (lane 1) and the absence of this integral protein of the endoplasmic reticulum in samples of isolated mitochondria from SH-SY5Y mock or APPswedish transfected cells (lanes 3–4). (**E**) Protein level of superoxide dismutase 2 (SOD2) in homogenate (lane 1) and in samples of isolated mitochondria from SH-SY5Y mock or APPswedish transfected cells (lanes 3–4). (**F**) Transmission electron microscopy of SH-SY5Y APPwt cells and isolated mitochondria. 1: A typical SH-SY5Y APP cell with mitochondria distributed throughout the cell at 2000× magnification. 2–3: 12,000× magnification of mitochondria from 1). 4–5: Mitochondrial fraction specifically isolated from SH-SY5Y APP cells at 12,000× magnification. Round structures in the range of 200–800 nm are visible, indicating a pure mitochondrial fraction without contamination with other cell organelles. The internal membrane structures of the mitochondria are largely collapsed due to preparation. Scale bars: 1: 1000 nm; 2–5: 500 nm.

To investigate whether lipid changes in total cell homogenates are comparable to mitochondria or might be organelle-specific, we isolated mitochondria of the human neuroblastoma cell line SH-SY5Y, stably transfected with the familial APPswedish mutation (SH-SY5Y APPswedish), leading to increased Aβ levels [34,35]. Lipid changes observed for this cellular AD model were compared to SH-SY5Y cells stably transfected with the expression vector alone as control (SH-SY5Y mock-transfected cells). Mitochondria were isolated as described in the materials and methods section and the purity of the isolated mitochondria fraction was analyzed by Western blot (WB) analysis utilizing the following specific organelle marker proteins: β-actin as cytoskeleton marker, early endosomal antigen 1 (EEA1) as marker for endosomes, LAMP1 (lysosomal-associated membrane protein 1) for lysosomes, calnexin as marker for the endoplasmic reticulum (ER) and SOD2 (superoxide dismutase 2) as marker for mitochondria. It has to be emphasized that purity of the mitochondria, and in particular, detaching the endoplasmic reticulum, was one of the main objectives, because lipid composition is highly dependent on the source of the analyzed membrane. As shown in Figure 1, β-actin was only detectable in total cell homogenates but not in the mitochondria fractions of mock-transfected or APPswedish transfected SH-SY5Y cells (Figure 1A). Similar to β-actin, we only obtained a WB signal for EEA1 (Figure 1B) and LAMP1 (Figure 1C) in total cell homogenate but not in the mitochondria fractions (Figure 1B,C), indicating that endosomes and lysosomes were appropriately separated from mitochondria. As the ER is covalently connected to mitochondria, the separation of mitochondria from the ER is one of the most difficult issues to gain mitochondria of high purity. By the use of proteinase K, we were able to detach the ER from the mitochondria. The ER marker calnexin (Figure 1D) was only detectable in total cell homogenates, whereas SOD2, the mitochondrial marker, could be detected in total cell homogenates as well as in the mitochondria fractions of mock-transfected as well as APPswedish transfected human neuroblastoma cells (Figure 1E). The purity of the isolated mitochondria was further analyzed by electron microscopy (EM) (Figure 1F). As in the EM images, solely mitochondria are found, this result further underlies the purity of the isolated mitochondria.

Before the analysis of in total 226 lipid species by targeted shotgun mass spectrometry (MS), cell homogenates and mitochondria fractions were adjusted to equal protein amount. No significant differences in the protein concentration of the mitochondria fractions in the APPswedish SH-SY5Y compared to mock-transfected cells were observed (*p* = 0.207). However, by protein adjustment, we wanted to exclude potential differences caused by deviations in the yield of the purification process. As mentioned in the introduction, we analyzed phosphatidylcholines, phosphatidylethanolamines as the major phospholipid species of mitochondria accompanied by the known AD-affected lipids corresponding plasmalogens and lyso-phospholipids and the acyl- and acetyl-carnitines to estimate an effect on mitochondrial function.

**Figure 2:**
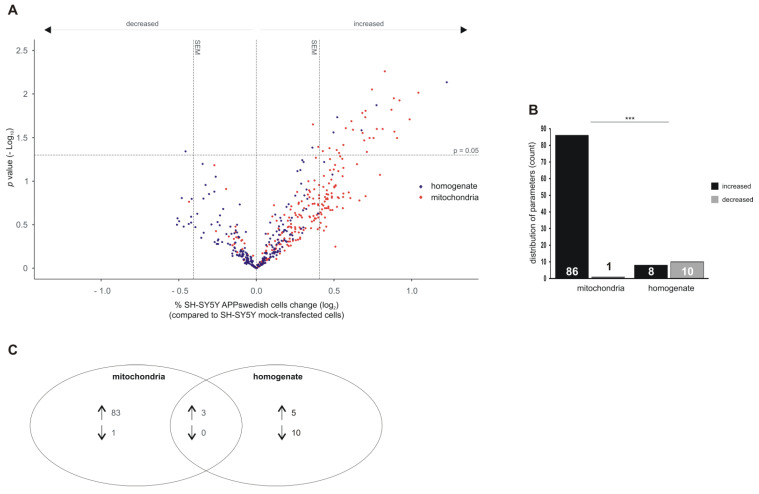
Lipid changes in homogenates and mitochondria of SH-SY5Y APPswedish cells in comparison to mock-transfected SH-SY5Y cells, respectively. (**A**) The fold changes of 226 lipid species were plotted logarithmically against the *p* value (-Log10) for homogenates (blue) and mitochondria (red). Vertical dashed lines next to 0 represent the average SEM and the horizontal dashed line marks *p* = 0.05. (**B**) Distribution of lipid species with a fold change greater than the average SEM and a *p* value ≤ 0.05 represented as number of decreased and increased lipid species in homogenate and mitochondria in a bar chart. Statistical significance was calculated using Fisher’s exact test with *** *p* ≤ 0.001. (**C**) Venn diagram showing the number of exclusively changed as well as overlapping lipid species in mitochondria and homogenates in SH-SY5Y APPswedish cells compared to mock-transfected cells. Arrows indicate decreasing (↓) or increasing (↑) effects.

These 226 lipid species were semi-quantitatively measured by target MS approach in total cell homogenates and mitochondria fractions of APPswedish transfected SH-SY5Y cells compared to total cell homogenates and mitochondria fractions of control cells (Figure 2A). Obtained data were normalized to deuterated standards added before lipid extraction to exclude effects of the lipid extraction and calculated as x-fold change compared to the control group. The volcano plot shown in Figure 2 show lipid changes in total cell homogenates (blue dots) and mitochondria (red dots) of the cellular AD model compared to control cells. Results are shown as volcano plots combining significance and effect strength.

We would like to emphasize that beside the statistically significant changes, we also focused on alterations of lipid species, which showed an effect strength higher than the average standard error of the mean (SEM). These changes were described as a trend. In addition, analyzing these trends allowed us to identify “group effects”, that means the distribution of lipids with similar chemical similarities showing all the same trend but without reaching significance for the individual lipid species. By just focusing on the significant lipid species, these group effects will be mainly unrecognized. Moreover, we would like to point out that the definition of significance has an arbitrary aspect being highly dependent on the statistical method and the number of samples. However, non-significant results should not be over interpreted and should be discussed carefully and need to be confirmed by other studies.

Interestingly, we found that in mitochondria of APPswedish transfected cells, 86 lipid species tended to increase, whereas only one species tended to decrease with an effect strength greater than the average standard error of the mean (the average SEM is presented in the volcano plot as vertical dashed lines next to 0). Of total cell homogenates of the cellular AD model, only eight lipid species tended to increase, whereas ten species tended to decrease (Figure 2B). The observed shift of up- and down-regulated lipid species in mitochondria compared to total cell homogenates was highly significant, indicating that specific mitochondrial lipid alterations exist. Indeed, within the 86 lipid species found to be increased in mitochondria of APPswedish transfected cells, 83 lipid species were specific for mitochondria, whereas only three of the up-regulated lipids were also found in total cell homogenate (Figure 2C). Five out of the eight lipid species found to be elevated in total cell homogenates were specific for total cell homogenate. The one lipid species trend to be decreased in mitochondria could be exclusively found in the mitochondria fractions and ten down-regulated lipid species could also be detected exclusively in total cell homogenate. These results indicate that the mitochondrial lipid composition differs at least partially from total cell homogenate and is analyzed in detail in the following paragraphs.

### 3.2. Mass Spectrometry Analysis of Phosphatidylcholine (PCaa) in Mitochondria and Total Cell Homogenate of APPswedish Transfected SH-SY5Y Cells

**Figure 3:**
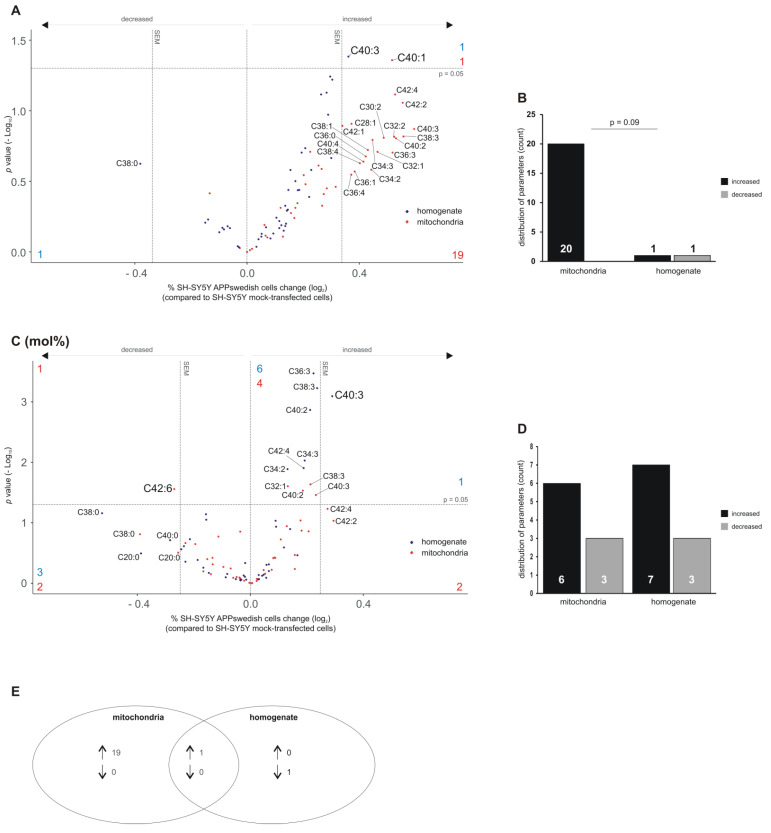
Changed phosphatidylcholine (PCaa) levels in homogenates and mitochondria of SH-SY5Y APPswedish cells in comparison to mock-transfected SH-SY5Y cells, respectively. (**A**,**B**) The fold changes of single PCaa species in homogenates and mitochondria are shown as volcano plots and the corresponding distribution analysis is shown in a bar chart on the right. (**C**,**D**) The effects of single PCaa species independent of PCaa lipid class effect are presented as appropriate volcano plot and bar chart. Vertical dashed lines next to 0.0 represent the average SEM and the horizontal dashed line marks *p* = 0.05 in volcano plots. Statistical significance was calculated using Fisher’s exact test. (**E**) Venn diagram showing the number of exclusively changed as well as overlapping PCaa species in mitochondria and homogenates in SH-SY5Y APPswedish cells compared to mock-transfected cells. Arrows indicate decreasing (↓) or increasing (↑) effects.

As mentioned above, phosphatidylcholine species (PCaa) represent beside phosphatidylethanolamine species (PEaa) the most prominent lipid species in mitochondria. We found that 20 out of 43 analyzed PCaa species (PCaa C28:1, C30:2, C32:1, C32:2, C34:2, C34:3, C36:0, C36:1, C36:3, C36:4, C38:1, C38:3, C38:4, C40:2, C40:3, C40:4, C42:1, C42:2, C42:4) tended to be increased in the mitochondria fraction (Figure 3A,B). One lipid species, PCaa C40:1, revealed a significant increase (significant lipid species are highlighted in the volcano plots above the horizontal dashed line with a larger font size) in APPswedish mitochondria compared to control (Figure 3B). It should be underlined that as a method-related caveat, the sum of the FAs bound to the sn-1 and sn-2 position is presented. Interestingly, the majority of the analyzed PCaa species revealed only small alterations within the average standard error of the mean in the total cell homogenates (Figure 3A). Only one PCaa species, PCaa C40:3, was significantly altered in homogenates of the cellular AD model compared to control cells. No trends of decreased PCaa species were found in mitochondria, and one lipid species (PCaa C38:0) showed a trend in cell homogenates (Figure 3B). The Venn diagram in Figure 3E illustrates that 19 out of the 20 up-regulated PCaa species were specific for mitochondria, whereas one PCaa species was found to be increased in both mitochondria fractions and total cell homogenate (Figure 3E), indicating that the APPswedish mediated changes in lipid composition in PCaa differs between mitochondria and total cell homogenate.

In Figure 3C, the distribution of the PCaa species independent of the total PCaa changes (changes in mol%) are shown. To analyze the distribution pattern of the lipid species within a lipid class, the lipid species signals were divided by the sum of lipid species of the lipid class. This result illustrates the percent of a single lipid species compared to its lipid class. Six PCaa species in mitochondria showed either a trend to be up-regulated (PCaa C42:2 and C42:3) or were significantly increased (PCaa C32:1, C38:3, C40:2, C40:3) (Figure 3C). Three PCaa species were found to be decreased in mitochondria: two PCaa species showed a trend (PCaa C20:0, C38:0), one species, PCaa C42:6, was significantly decreased (Figure 3C,D). Seven PCaa species were significantly up-regulated in total cell homogenates of APPswedish transfected cells compared to control cells (PCaa C34:2, C34:3, C36:3, C38:3, C40:2, C40:3, C42:4) (Figure 3C). Notably, PCaa C40:3 was significantly altered in total cell homogenate, both in mol% (Figure 3C) or data not normalized to total PCaa content (Figure 3A).

### 3.3. Alterations in Phosphatidylcholine Plasmalogen Species (PCae) in Mitochondria and Total Cell Homogenate of SH-SY5Y Cells Stably Expressing APPswedish

**Figure 4:**
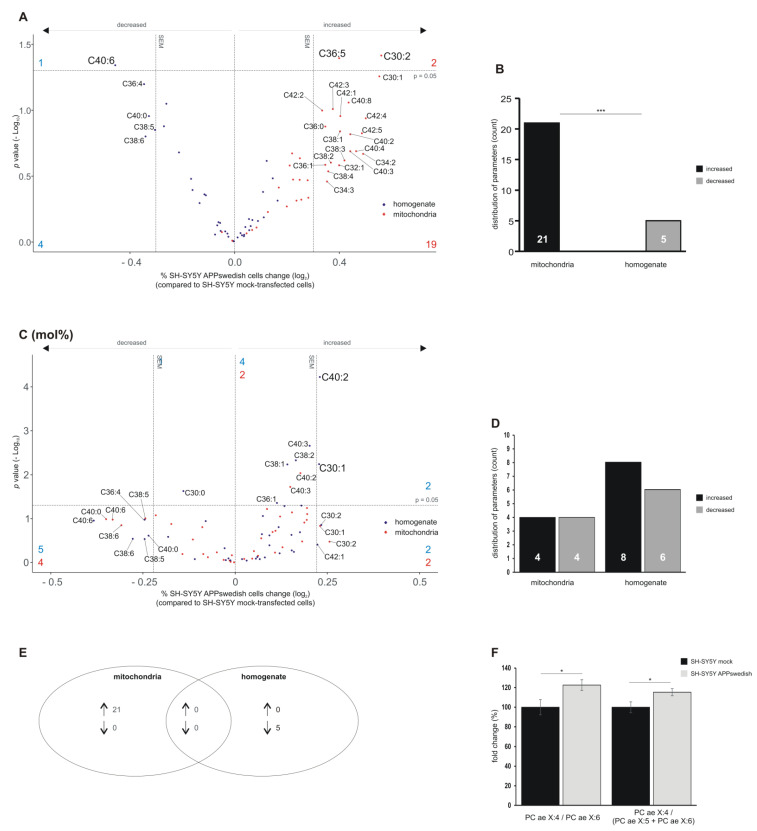
Changed phosphatidylcholine-plasmalogens (PCae) levels in homogenates and mitochondria of SH-SY5Y APPswedish cells in comparison to mock-transfected SH-SY5Y cells, respectively. (**A**,**B**) The fold changes of single PCae species in homogenates and mitochondria are shown as volcano plots and the corresponding distribution analysis is shown in a bar chart on the right. (**C**,**D**) The effects of single PCae species independent of PCae lipid class effect are presented as appropriate volcano plot and bar chart. Vertical dashed lines next to 0.0 represent the average SEM and the horizontal dashed line marks *p* = 0.05 in volcano plots. Statistical significance was calculated using Fisher’s exact test with *** *p* ≤ 0.001. (**E**) Venn diagram showing the number of exclusively changed as well as overlapping PCae species in mitochondria and homogenates in SH-SY5Y APPswedish cells compared to mock-transfected cells. Arrows indicate decreasing (↓) or increasing (↑) effects. (**F**) Fold changes of PCae X:4/PCae X:6- and PCae X:4/(PCae X:5 + PCae X:6) ratios, representing the ω3/ω6 relation, in mitochondria samples of SH-SY5Y APPswedish cells compared to SH-SY5Y mock-transfected cells. Statistical significance was calculated using two-tailed Student’s *t*-test with * *p* ≤ 0.05.

Plasmalogens known to be altered in AD are characterized by an enolether at the sn-1 position of the glycerol backbone, making plasmalogens more susceptible to oxidative stress than the corresponding ester-bonded glycerophospholipid. Similar to PCaa species, we found that the majority of the PCae species tended to be increased in mitochondria. Of the 39 analyzed PCae species, 21 revealed a trend (PCae C30:1, C32:1, C34:2, C34:3, C36:0, C36;1, C38:1, C38:2, C38:3, C38:4, C40:2, C40:3, C40:4, C40:8, C42:1, C42:2, C42:3, C42:4, C42:5); two of them (PCae C36:5 and PCae C30:2) showed a significant increase (Figure 4A,B). In contrast, in total cell homogenates, no PCae species tended or were significantly increased, but five PCae species showed a trend to be decreased (PCae C36:4, C38:5, C38:6, C40:0) (Figure 4A,B). Interestingly, another lipid species having six times the unsaturated fatty acids was found to be significantly decreased in cell homogenates (PCae C40:6). The differences in PCae species to be up-regulated in mitochondria and to be down-regulated in total cell homogenate was highly significant (Figure 4B). Twenty-one up-regulated PCae species found in the mitochondria fractions were specific for mitochondria and the five down-regulated PCae species found in total cell homogenates were specific for homogenate (Figure 4D). Analysis of the PCae mol% content revealed that two PCae species showed a trend to be increased in mitochondria (PCae C30:1 and C30:2); two PCae species showed significant alterations (PCae C40:2 and C40:3) (Figure 4C). Four PCae species showed a trend to be decreased (PCae C38:5, C38:6, C40:0, C40:6) (Figure 4C,D). For total cell homogenates, two PCae species tended to be increased (PCae C30:2, C42:1) and six species were significantly elevated (PCae C30:1, C36:1, C38:1, C38:2, C40:2, C40:3) (Figure 4D,E). Five out of the 39 analyzed PCae species showed a trend to be decreased (PCae C36:4, C38:5, C38:6, C40:0, C40:6) and PCae C30:0 was significantly reduced (Figure 4C,D). Further evaluation and processing of the obtained data for mitochondria revealed that the ratio of PCae X:4/PCae X:6 is significantly increased in APPswedish transfected SH-SY5Y cells (122.7% ± 5.5%, *p* ≤ 0.05) (Figure 4F). These PCae species might include arachidonic acid (C20:4) (an omega-6 FA) and docosahexaenoic acid (C22:6) (an omega-3 FA), both discussed to be affected in AD [47,54,55]. Similarly, the ratio of PCae X:4/PCae X:5 + PCae X:6 is significantly increased in the cellular AD model (115.3% ± 3.7%, *p* ≤ 0.05) (Figure 4F), suggesting that the omega-3 FAs docosahexaenoic acid and eicosapentaenoic acid (C20:5) are decreased in mitochondria of APPswedish transfected cells.

### 3.4. Lyso-Phosphatidylcholine (Lyso-PC) Species

**Figure 5:**
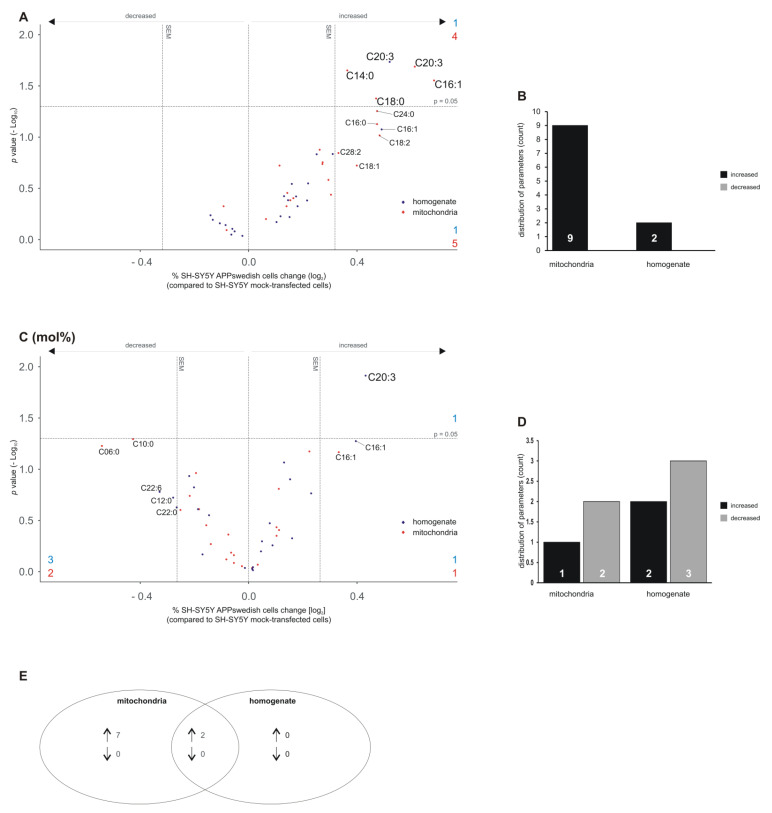
Changed lyso-phosphatidylcholine (lyso-PC) levels in homogenates and mitochondria of SH-SY5Y APPswedish cells in comparison to mock-transfected SH-SY5Y cells, respectively. (**A**,**B**) The fold changes of single lyso-PC species in homogenates and mitochondria are shown as volcano plots and the corresponding distribution analysis is shown in a bar chart on the right. (**C**,**D**) The effects of single lyso-PC species independent of lyso-PC lipid class effect are presented as appropriate volcano plot and bar chart. Vertical dashed lines next to 0.0 represent the average SEM and the horizontal dashed line marks *p* = 0.05 in volcano plots. Statistical significance was calculated using Fisher’s exact test. (**E**) Venn diagram showing the number of exclusively changed as well as overlapping lyso-PC species in mitochondria and homogenates in SH-SY5Y APPswedish cells compared to mock-transfected cells. Arrows indicate decreasing (↓) or increasing (↑) effects.

Analyzing lyso-PC species in mitochondria fractions and total cell homogenates revealed that some lyso-PC species showed a trend to be increased in both samples whereas none of the 22 analyzed lyso-PC species showed a trend to be decreased (Figure 5A). Five lyso-PC species showed a trend to be elevated in mitochondria (lyso-PC C16:0, C18:1, C18:2, C24:0, C28:2); four increased lyso-PC species showed a significant effect: lyso-PC C14:0, C18:0, C16:1 and C20:3 (Figure 5A,B). One lyso-PC species revealed a trend to be elevated in total cell homogenate (lyso-PC C16:1) and one lyso-PC species, lyso-PC C20:3, showed a significant effect (Figure 5A,B). Interestingly, the two lyso-PC species found to be elevated in total cell homogenate were also found to be up-regulated in the mitochondria fractions (Figure 5E). Analysis of the mol% of the lyso-PC showed lyso-PC C20:3 also to be significantly increased in total cell homogenate (Figure 5C). A second lyso-PC species showed a trend to be up-regulated (lyso-PC C16:1), whereas three lyso-PC species (lyso-PC C12:0, C20:0, C22:6) tended to be decreased in total cell homogenates when normalized to total lyso-PC content (Figure 5D). For mitochondria, one lyso-PC species tended to be elevated (lyso-PC C16:1) and two lyso-PC species tended to be decreased in lyso-PC mol% (lyso-PC C06:0 and C10:0) (Figure 5C,D).

### 3.5. Alterations in Phosphatidylethanolamine Species (PEaa) in Mitochondria and Total Cell Homogenate of APPswedish Transfected Cells

**Figure 6:**
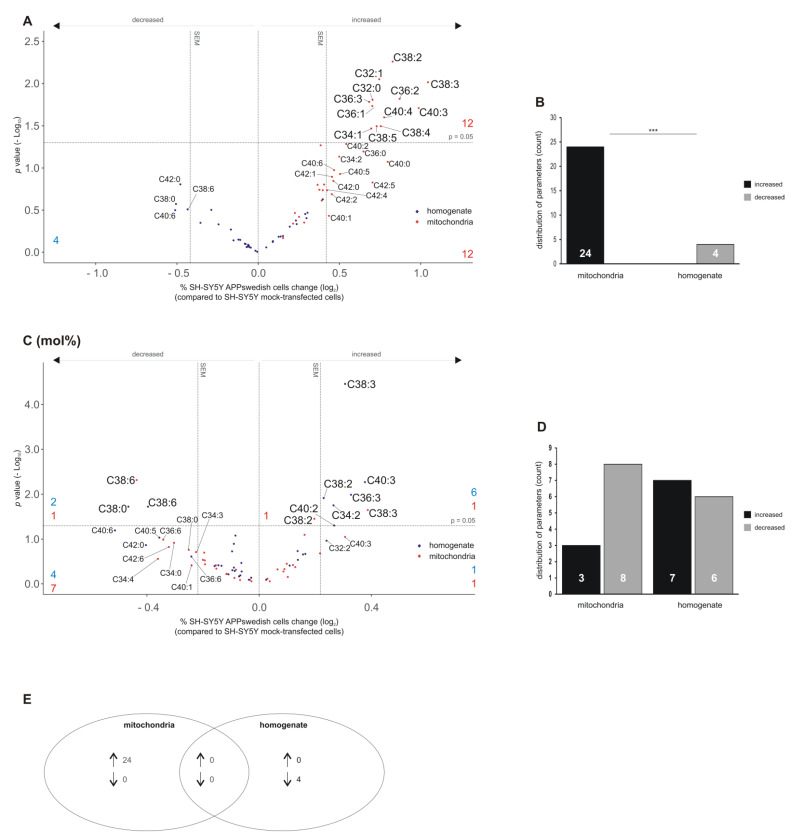
Changed phosphatidylethanolamine (PEaa) levels in homogenates and mitochondria of SH-SY5Y APPswedish cells in comparison to mock-transfected SH-SY5Y cells, respectively. (**A**,**B**) The fold changes of single PEaa species in homogenates and mitochondria are shown as volcano plots and the corresponding distribution analysis is shown in a bar chart on the right. (**C**,**D**) The effects of single PEaa species independent of PEaa lipid class effect are presented as appropriate volcano plot and bar chart. Vertical dashed lines next to 0.0 represent the average SEM and the horizontal dashed line marks *p* = 0.05 in volcano plots. Statistical significance was calculated using Fisher’s exact test with *** *p* ≤ 0.001. (**E**) Venn diagram showing the number of exclusively changed as well as overlapping PEaa species in mitochondria and homogenates in SH-SY5Y APPswedish cells compared to mock-transfected cells. Arrows indicate decreasing (↓) or increasing (↑) effects.

Similar to the results obtained for the analysis of PCaa species, we found that twelve out of the 35 analyzed PEaa species tended to be increased (PEaa C34:2, C36:0, C40:0, C40:1, C40:5, C40:2, C40:6, C42:0, C42:1, C42:2, C42:4, C42:5), whereas no PEaa species tended to be decreased in the mitochondria fractions (Figure 6A,B). Of the 24 elevated PEaa species in mitochondria, 12 revealed a significant effect: PEaa C32:0, C32:1, C34:1, C36:1, C36:2, C36:3, C38:2, C38:3, C38:4, C38:5, C40:3 and C40:4 (Figure 6A). These PEaa species mainly include “midchain” fatty acids (C16-C20). Moreover, in line with the findings of PCaa species, no PEaa species were found to be increased in total cell homogenate, but four PEaa species showed a trend to be decreased (PEaa C38:0, C38:6, C40:6, C42:0) (Figure 6A,B). The observed differences in up- and down-regulation of PEaa species were highly significant between mitochondria and total cell homogenate (Figure 6B,E).

Mol% PEaa analysis revealed that PEaa C38:6 was significantly reduced in both mitochondria fractions as well as total cell homogenate (Figure 6C). Notably, this lipid species might include the combination of FAs C16:0 and docosahexaenoic acid C22:6 or C18:1 and eicosapentaenoic acid 20:5. Reductions in these lipid species are known to be associated with AD. This finding is in line with our results showing an elevated ratio of X:4/X:5 phosphatidylethanolamine plasmalogens in mitochondria of the cellular AD model. Beside PEaa C38:6, PEaa C38:0 was significantly reduced in total cell homogenate of APPswedish transfected cells. PEaa C38:3 that has been found to be significantly increased in mitochondria fractions was also significantly elevated in PEaa mol% (Figure 6C).

For total cell homogenates, we found six PEaa species to be significantly increased in PCae mol%: PEaa C34:2, C36:3, C38:2, C38:3, C40:2 and C40:4 (Figure 6C). Moreover, PEaa C32:2 tended to be elevated (Figure 6C). In total, one PEaa species tended to be increased in mitochondria (PEaa C40:3) and two PEaa species were significantly increased (PEaa C38:2 and C38:3) (Figure 6C,D). Eight PEaa species were decreased, the DHA or EPA-containing PEaa C38:6 being significant, and seven PEaa species showed a trend to be decreased (PEaa C34:0, C34:3, C34:4, C36:3, C38:0, C40:1, C42:6). For total cell homogenate, we found that one PEaa species tended to be elevated (PEaa C32:2) and six PEaa species (PEaa C34:2, C36:3, C38:2, C38:3, C40:2, C40:3) tended to be significantly decreased in PEaa mol% (Figure 6D).

### 3.6. Analysis of Phosphatidylethanolamine Plasmalogens (PEae) in Mitochondria and Total Cell Homogenates of SH-SY5Y APPswedish Cells

**Figure 7:**
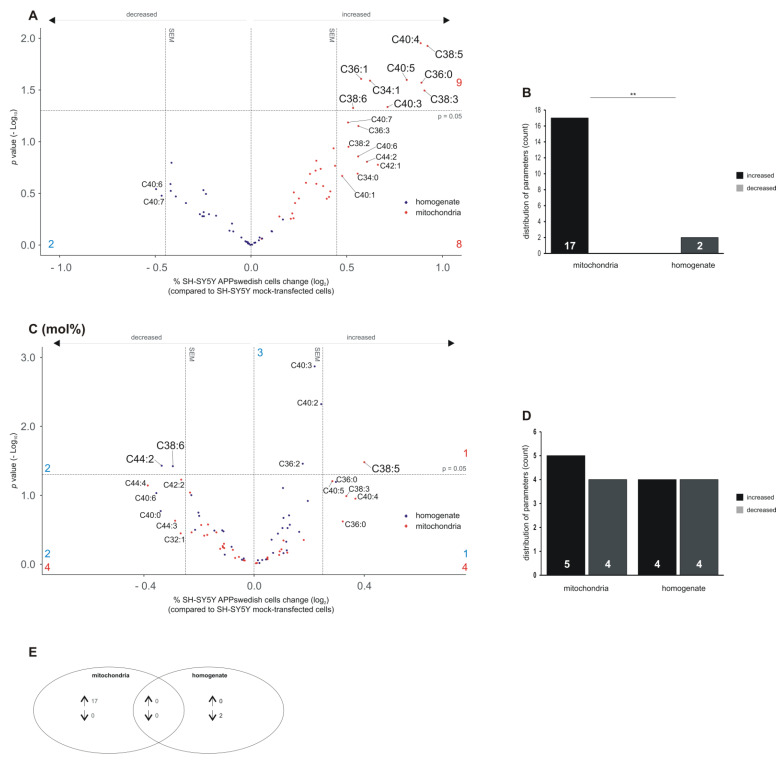
Changed phosphatidylethanolamine plasmalogens (PEae) levels in homogenates and mitochondria of SH-SY5Y APPswedish cells in comparison to mock-transfected SH-SY5Y cells, respectively. (**A**,**B**) The fold changes of single PEae species in homogenates and mitochondria are shown as volcano plots and the corresponding distribution analysis is shown in a bar chart on the right. (**C**,**D**) The effects of single PEae species independent of PEae lipid class effect are presented as appropriate volcano plot and bar chart. Vertical dashed lines next to 0.0 represent the average SEM and the horizontal dashed line marks *p* = 0.05 in volcano plots. Statistical significance was calculated using Fisher’s exact test with ** *p* ≤ 0.01. (**E**) Venn diagram showing the number of exclusively changed as well as overlapping PEae species in mitochondria and homogenates in SH-SY5Y APPswedish cells compared to mock-transfected cells. Arrows indicate decreasing (↓) or increasing (↑) effects.

As illustrated in the volcano plot in Figure 7A, all analyzed PEae species tended to be elevated in mitochondria, whereas the majority of the PEae species tended to be decreased in total cell homogenate (Figure 7A). Nine out of the seventeen elevated PEae species were significantly changed: PEae C34:1, C36:0, C36:1, C38:3, C38:5, C38:6, C40:3, C40:4 and C40:5. Eight PEae species showed a trend to be elevated in mitochondria (PEae C34:0, C36:3, C38:2, C40:1, C40:6, C40:7, C42:1, C44:2). Two PEae species (PEae C40:6, C40:7) showed a trend to be decreased in total cell homogenate (Figure 7C). The observed differences in elevated and decreased PEae species were highly significant between mitochondria and total cell homogenate, which is in line with the results obtained for PCae species, where the alterations between mitochondria and total cell homogenate show a high significance (see Figure 4B).

Analyzing the PEae mol%, the distribution of up- and down-regulated PEae species seems to be similarly disseminated between mitochondria and total cell homogenate (Figure 7C,D). Four PEae species (PEae C36:0, C38:3, C40:4, C40:5) tended to be increased in mitochondria fractions by normalization to total PEae content and one PEae species, PEae C38:5 revealed a significant increase (Figure 7C). Notably, PEae C38:5 has already been found to be significantly up-regulated in mitochondria in unprocessed data. Four PEae species (PEae C32:1, C42:2, C44:3, C44:4) tended to be decreased in the mitochondria fractions (Figure 7C,D). A similar distribution of decreased and increased species was found for total cell homogenate: one PEae species (PEae C36:0) tended to be increased and three species are elevated significantly (PEae C36:2, C40:2, C40:3). Two PEae species tended to be decreased (PEae C40:0, C40:6) and PEae C38:6 and C44:2 revealed a significant effect (Figure 7D,E).

Notably, in the distribution of PEaa (mol%), PEaa C38:6 already revealed a significant reduction in cell homogenates of APPswedish transfected cells (see Figure 6C). Furthermore, it has to be mentioned that several of the significantly altered phosphatidylethanolamine plasmalogens (PEae C34:1, C36:1, C38:3, C38:5, C40:3, C40:4) in mitochondria have already been found to be significantly increased in the corresponding PEaa glycerophospholipid (PEaa C34:1, C36:1, C38:3, C38:5, C40:3, C40:4). The heat map shown in Supplemental Figure S4A further illustrates the observed effects between single lipid species in mitochondria and total cell homogenate combining all lipid classes (PC, PE, plasmalogen (ae) or esther bound fatty acid (aa)) (Supplemental Figure S4A).

### 3.7. Alterations in Lyso-Phosphatidylethanolmine (lyso-PE) Species in Mitochondria and Total Cell Homogenate of APPswedish Transfected SH-SY5Y Cells

**Figure 8:**
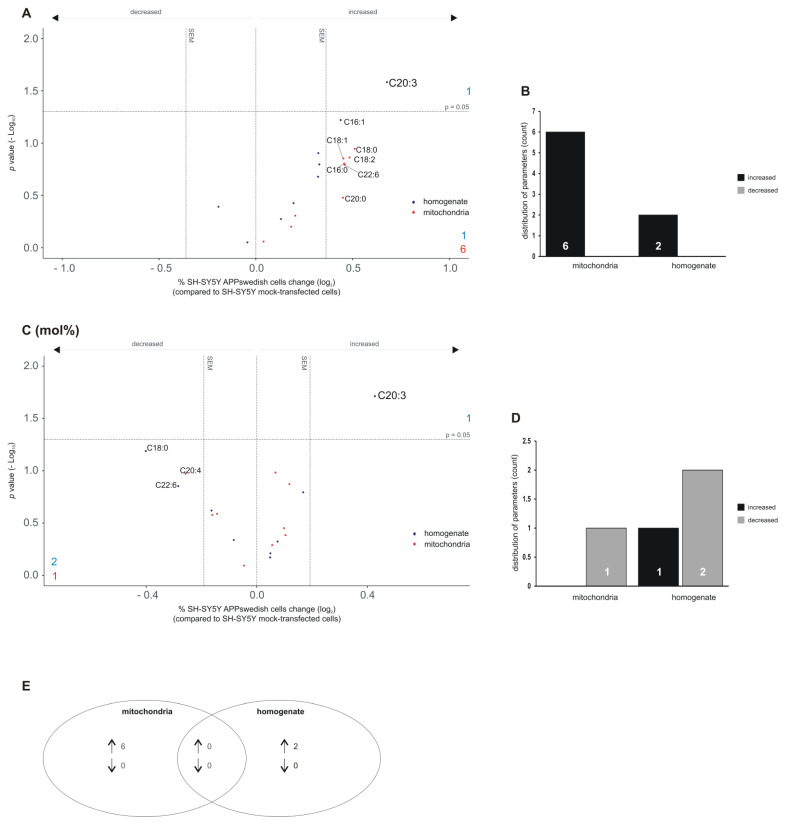
Changed lyso-phosphatidylethanolamine (lyso-PE) levels in homogenates and mitochondria of SH-SY5Y APPswedish cells in comparison to mock-transfected SH-SY5Y cells, respectively. (**A**,**B**) The fold changes of single lyso-PE species in homogenates and mitochondria are shown as volcano plots and the corresponding distribution analysis is shown in a bar chart on the right. (**C**,**D**) The effects of single lyso-PE species independent of lyso-PE lipid class effect are presented as appropriate volcano plot and bar chart. Vertical dashed lines next to 0.0 represent the average SEM and the horizontal dashed line marks *p* = 0.05 in volcano plots. Statistical significance was calculated using Fisher’s exact test. (**E**) Venn diagram showing the number of exclusively changed as well as overlapping lyso-PE species in mitochondria and homogenates in SH-SY5Y APPswedish cells compared to mock-transfected cells. Arrows indicate decreasing (↓) or increasing (↑) effects.

The analysis of lyso-PE species revealed that six out of the nine analyzed lyso-PE species tended to be increased in mitochondria of APPswedish transfected cells (lyso-PE C16:0, C18:0, C18:1, C18:2, C20:0, C22:6) (Figure 8A,B). These elevated lyso-PE species were specific for mitochondria (Figure 8E). None of the analyzed lyso-PE species tended to be decreased in mitochondria (Figure 8A,B). For total cell homogenate, one lyso-PE species tended to be elevated (lyso-PE C16:1) and lyso-PE C20:3 reached a significant effect (Figure 8A,B).

Similar to mitochondria, no lyso-PE species tended to be decreased (Figure 8A,B) in total cell homogenate. Interestingly, lyso-PE C20:3 mol% was also significantly increased in cell homogenate (Figure 8C). Two lyso-PE species in homogenate (lyso-PE C18:0, C22:6) and one in mitochondria (lyso PE C20:4) tended to be decreased in lyso-PE mol% (Figure 8D). Remarkably, identical to lyso-PE C20:3, lyso-PC 20:3 was already significantly elevated in cell homogenate, both in mol% or not normalized (see Figure 5A,C). The heat map in Supplemental Figure S4B further illustrates the observed effects of lyso-PC and lyso-PE species in mitochondria and cell homogenates of both PC and PE lyso species (Supplemental Figure S4B).

### 3.8. Analysis of the Carnitine Carrier System in Total Cell Homogenate

**Figure 9:**
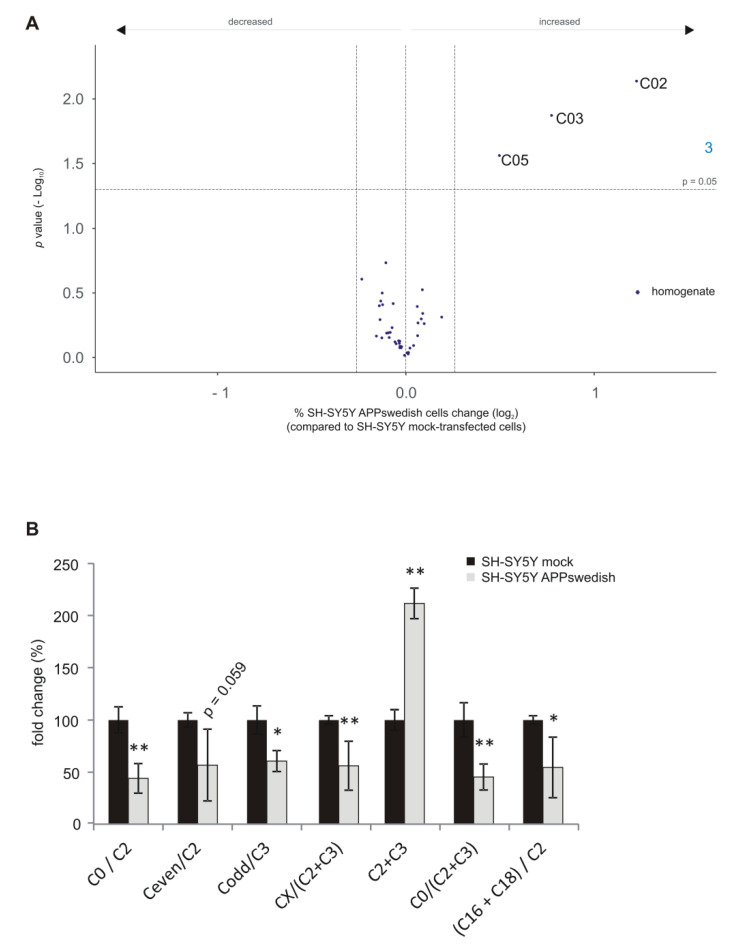
Changed carnitine levels in homogenates of SH-SY5Y APPswedish cells in comparison to mock-transfected SH-SY5Y cells. (**A**) The fold changes of single carnitine species in homogenates are shown as volcano plots. Vertical dashed lines next to 0.0 represent the average SEM and the horizontal dashed line marks *p* = 0.05. (**B**) The fold changes of C0/C2−, Ceven/C2−, Codd/C3−, CX/(C2 + C3)−, C2 + C3−, C0/(C2 + C3)− and (C16 + C18)/C2 ratios in SH-SY5Y APPswedish cells compared to SH-SY5Y mock cells are shown as bar charts. Statistical significance was calculated using two-tailed Student’s *t*-test with * *p* ≤ 0.05 and ** *p* ≤ 0.01.

As mentioned above, we also analyzed the carnitine carrier system to evaluate potential changes in mitochondrial function. To address this question, we analyzed 19 acyl-/acetyl-carnitine species in total cell homogenate of APPswedish transfected SH-SY5Y cells compared to control cells. Three out of the nineteen analyzed carnitine species revealed a significant increase: C02, C03 and C05 (Figure 9A). C02 and C03 represent degradation products that occur after mitochondrial β-oxidation of FAs bound to carnitine. In line, compared to mock transfected cells the sum of C2 + C3 was also significantly elevated in the cellular AD model (Figure 9B). In contrast, the ratio of C0 (carnitine with no bound fatty acid)/C2, Ceven/C2, Codd/C3, CX/(C2 + C3), C0/(C2 + C3) and (C16 + C18)/C2 were significantly decreased. These changes indicate alterations in β-oxidation and/or transport of the carnitine carrier system in the cellular AD model. As glucose metabolism has been reported to be decreased in AD [56,57,58], β-oxidation of FAs might be compensatory up-regulated, leading to elevated degradation products of β-oxidation in the APPswedish cell model. However, at least for familial presenilin mutations, a decrease in β-oxidation was found [26], pointing more to an alteration in the shuttle system of the carnitine carrier system. Regardless of the causal reason for the changes in the acyl-/acetyl-carnitines (β-oxidation or carnitine carrier system), it can be stated that the mitochondrial function is affected by the expression of the AD relevant APPswedish mutation.

### 3.9. Analysis of Cardiolipin and Phosphatidyl-Glycerol in Total Cell Homogenate

Several lines of evidence indicate that cardiolipin is strongly associated with neurological disorders, in particular, with AD [59]. Therefore, we would like to point out that the following results are not completely new investigations, but are important to evaluate whether our cell culture system results in similar cardiolipin changes found in other AD model systems or patients. As discussed later in detail, cardiolipin has been shown to be decreased in AD [60,61]. This is in line with our results showing a 26.7% decrease in total cardiolipin in the APPswedish transfected cells compared to mock transfected cells measured by a fluorescence-based assay (Supplemental Figure S6). A fluorescence-based assay has been chosen to analyze cardiolipin to ensure that all cardiolipin species are included. In the case of cardiolipin, this is of particular importance as cardiolipin has a large mol weight due to four fatty acids and not many species can be detected clearly without any impact of isotopic overlay or impact of isobaric masses without corrections, e.g., described in [62] but which would be beyond the scope of this article. However, it has to be emphasized that several studies also indicate that the fatty acid composition of cardiolipin is changed in AD [61]. To take this point into account and to add additional new data in respect to a potentially changed cardiolipin profile in AD, we have analyzed phosphatidyl-glycerol (PG). PG is a precursor of cardiolipin and therefore, it is reasonable to assume that a potentially changed cardiolipin profile is reflected by alterations in the PG profile (Supplemental Figure S7A). Interestingly, in particular, PG species with FAs mainly found in cardiolipin [63] are altered (PG 18:2/16:0; PG 18:2/16:1; PG 18:2/18:1, PG 18:2/18:2; PG 18:2/20:3; PG 18:2/20:4) (Supplemental Figure S7B,C). Again, this is in line with previous reports showing a changed cardiolipin profile in AD. Nevertheless, it should be pointed out that this is an indirect approach and further studies are needed to evaluate the above-mentioned results.

### 3.10. Alterations in Mitochondrial Lipid Composition of the Cellular AD Model Compared to Neuroblastoma Cells Expressing Wildtype APP

As mentioned in the introduction, APP can be processed via amyloidogenic and non-amyloidogenic pathways leading to different products or gene regulation. Whereas APPswedish is mainly cleaved by β-secretase in the amyloidogenic pathway, APP is processed both by amyloidogenic and non-amyloidogenic pathways [64]. However, APP overexpression is also often used as a cellular model for AD. Keeping in mind that the differences between these two cellular models exist, we also analyzed mitochondria fractions of SH-SY5Y cells stably expressing APPwt and compared the alteration in lipid homeostasis to APPswedish transfected cells. As the major objective of this paper was to elucidate the lipid alteration in mitochondria, we focused, in this second cellular model, only on lipid alterations found in mitochondria.

As shown in Supplemental Figure S5A, PCaa species also tended to be increased in mitochondria of APPwt transfected cells compared to control cells. No significant differences in the effect strength were observed between APPswedish and APPwt expressing SH-SY5Y cells of these PCaa species (Supplemental Figures S1A and S5A ). Similarly, PCae species tended to be elevated in APPwt transfected cells compared to control. For some PCae species, the effect was not as pronounced; however, no significant differences were observed between APPwt and APPswedish transfected cells (Supplemental Figures S1A and S5B).

In line with the other PC lipid classes, the seven lyso-PC species that have already been found to be exclusively elevated in mitochondria of APPswedish cells also revealed an increase in mitochondria of APPwt expressing SH-SY5Y cells (Supplemental Figures S1A and S5C).

Interestingly, for all PE lipid classes, including phosphatidylethanolamine plasmalogens and lyso-phosphatidylethanolamines, we observed the most pronounced differences between APPswedish and APPwt expressing cells. The PEaa species that have been exclusively found to be elevated in mitochondria (and not homogenate) of APPswedish cells were only slightly elevated or even decreased in APPwt expressing cells (Supplemental Figures S1B and S5D). Significant differences between APPwt and APPswedish were observed for PEaa C32:0, PEaa C36:3, PEaa C40:2, PEaa C42:0 and PEaa C42:4 (Supplemental Figure S3).

A similar trend was found for phosphatidylethanolamine-plasmalogens. Comparable to PEaa, the PEae species found to be increased in mitochondria of APPswedish expressing cells only revealed a slight increase or were decreased compared to control cells in APPwt expressing cells (Supplemental Figures S1B and S5E). Comparing all PEae species between the two AD models, significant effects were observed for PEae C36:3, PEae C40:2 and PEae C44:5 (Supplemental Figure S3).

The six lyso-PE species detected to be exclusively elevated in mitochondria of APPswedish transfected cells compared to mitochondria of control cells were not altered in APPwt cells (Supplemental Figures S1B and S5F), also indicating that the most obvious differences between swedish and wt expressing cells exist in phosphatidylethanolamine lipid species.

In line with the PC species, the carnitine carrier system revealed no significant differences between the two AD cellular models, further indicating that PE-related lipid classes might be most specifically regulated depending on amyloidogenic or non-amyloidogenic APP processing (Supplemental Figure S5G).

## 4. Discussion

Mitochondrial dysfunction is a common characteristic of many neurodegenerative disorders, including AD and Parkinson’s disease, that are characterized by deposition of aggregated proteins as insoluble fibrils or plaques [65,66]. Mitochondrial function depends on the lipid environment to obtain mitochondrial fission and fusion, oxidative phosphorylation and mitochondrial bioenergetics [67]. In line, the recruitment and activity of several mitochondrial proteins is also dependent on the lipid environment, indicating that changes in mitochondrial lipid membrane composition might contribute to mitochondrial dysfunction and cell death associated with AD. Extensive lipid alterations have been reported in brains of AD-affected individuals and transgenic AD mouse models, including decreased plasmalogen and lyso-PC levels [38,39,40,41,42,44,45,46]. Vice versa, several lipids have been shown to alter Aβ level, either by affecting proteolytic processing of APP or by influencing Aβ degradation [43,52,68,69,70,71,72]. Increasing evidence suggests an important impact of omega-3 and omega-6 polyunsaturated fatty acids (PUFAs) in AD pathogenesis. The omega-3 fatty acids docosahexaenoic acid (DHA) and eicosapentaenoic acid (EPA) have been reported to decrease Aβ level by an elevation of Aβ degradation and by affecting APP processing leading to Aβ [52,68]. On the other hand, the omega-6 PUFA arachidonic acid is discussed to be increased in AD [47,54,55], leading to inflammatory processes. Based on the evidence of altered lipid homeostasis and mitochondrial dysfunction in AD, that might depend on a changed mitochondrial membrane lipid composition, lipid alterations in mitochondria of human neuroblastoma cells stably expressing the familial APPswedish mutation were analyzed and compared to lipid changes in total cell homogenate by a target lipidomics approach in order to investigate whether potential changes in lipid alterations are specific for mitochondria.

In our study, we focused on lipid classes mainly found in mitochondria, including PG, which is a precursor for cardiolipin. Besides these major lipid classes, obviously other lipids exist which are also important to understand potential changes in the mitochondrial function. Further studies are needed to address and analyze these more minor mitochondrial lipid species. Without knowing every lipid species, the effect of, e.g., the physical properties of the membranes are hard to estimate. This should be clearly pointed out as a caveat of our study. However, PC, PE and cardiolipin are reported to represent the main part of the mitochondrial membrane with over 90% [73].

It has to be pointed out that the results obtained by targeted shotgun mass spectrometry are semi-quantitative and have been normalized to deuterated standards added before lipid extraction to avoid inaccuracies caused by deviation in the yield of lipid extraction or ionization efficiency. As a potential caveat, matrix effects have to be taken into consideration. In principle, it cannot be ruled out that lipid species of different lipid classes affect each other, particularly if no HPLC separation of the lipid classes were performed. However, the impact of matrix effects on our results were on average below 2% (see Supplemental Figure S2; average matrix effect 1.19%). The results were presented as volcano plots for each lipid class combining effect strength and significance and we focused on results being significant or having an effect strength above the average standard error of the mean.

Based on our results, we could show that several lipid changes found for the SH-SY5Y APPswedish AD cellular model are specific for mitochondria and differ from total cell homogenate. Significant differences were observed in the number of increased and decreased phosphatidylcholine plasmalogens and phosphatidylethanolamine plasmalogens, revealing an increase for mitochondria and a decrease for total cell homogenate. In line, the number of phosphatidylcholine and phosphatidylethanolamine species to be increased with an effect strength greater than the average standard error of the mean or being significant was elevated in mitochondria. The observed reduction in plasmalogens in total cell homogenate of APPswedish transfected cells are in line with current literature, reporting reduced plasmalogen levels in brain and CSF of AD patients [38,39,74,75,76]. Besides the observed differences between mitochondria fractions and total cell homogenate, changes in lipid species such as specific plasmalogens (PCae and PEae) and phospholipids (PCaa and PEaa) or lyso-phospholipids also exist that are similarly altered in mitochondria and homogenate. For example, we observed an elevation of lyso-phospholipids (lyso-PC and lyso-PE) for both samples and lyso-PC C20:3 was significantly increased in mitochondria and cell homogenate of APPswedish expressing SH-SY5Y cells.

In line with previous literature and to evaluate our cell culture system, we found a decreased total cardiolipin level [59,60,61]. Cardiolipin is beside PC and PE a major lipid and accounts for 10–15% of mitochondrial lipid content, being primarily localized in the inner membrane of mitochondria [73,77]. Several roles of cardiolipin have been reported in particular in membrane architecture, mitophagy, mitochondrial dynamics, bioenergetics and protein import (e.g., reviewed in [59]). Therefore, cardiolipin has an indisputable role in mitochondrial function and energy homeostasis. AD is accompanied by an increase in reactive oxidative species (ROS). In respect to AD, an increase in ROS has been reported [78,79]. An increased ROS level results in oxidized cardiolipin which favors the release of apoptotic factors such as cytochrome C from the mitochondrial membrane to the cytosol [80]. Consequently, several studies found a positive effect on neuronal survival in AD models by increasing cardiolipin, e.g., by adding wheat germ agglutinin-conjugated liposomes incorporated with cardiolipin [81]. Moreover, intraperitoneal injections of liposomes containing besides phosphatidic acid cardiolipin have been shown to reduce amyloid-β levels in APP/PS1 transgenic mice [82]. Beside the known total decrease in cardiolipin which was also found in our AD model, an altered FA composition of cardiolipin has been reported and found to be associated with AD [61]. As already mentioned in the result section, we evaluated if these known changes in cardiolipin composition are also reflected by an altered FA composition in the precursor of cardiolipin, PG. Indeed, we found that particular PG species containing FAs dominantly found in cardiolipin were changed in APPswedish transfected cells compared to mock transfected cells [61]. This is in line with studies, e.g., showing that in 3xtransgenic AD mice, a change in cardiolipin profile is associated to the early synaptic mitochondrial dysfunction in AD [60].

**Figure 10:**
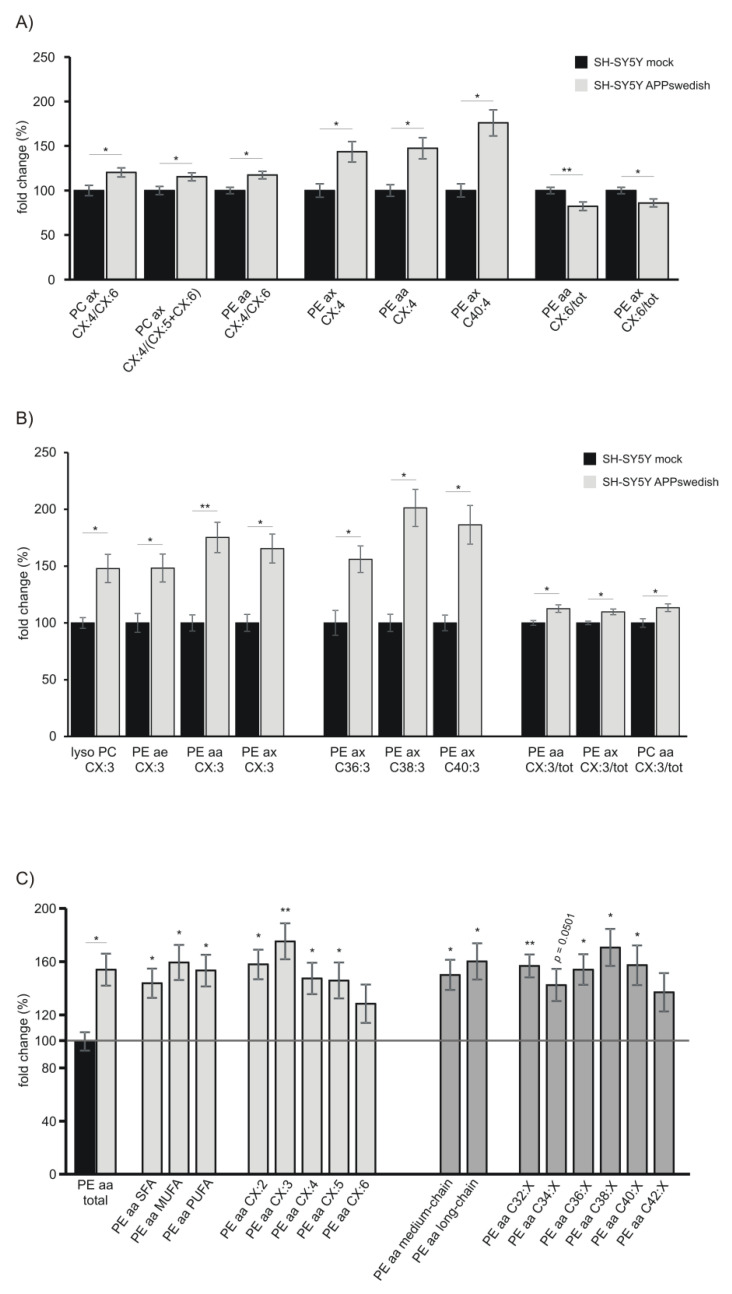

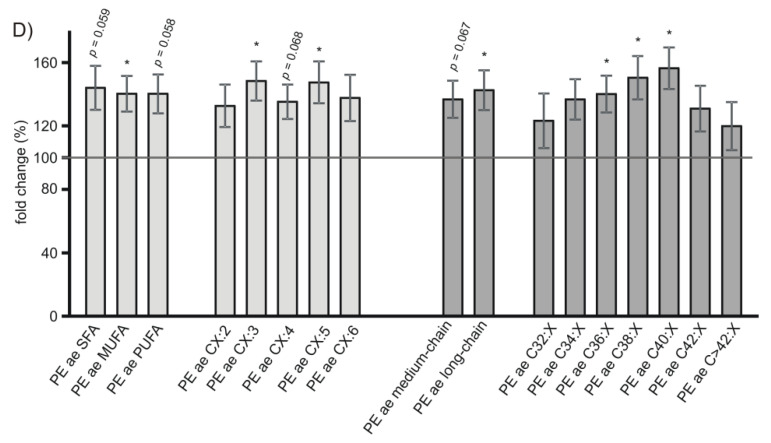
Processed data. (**A**) Fold changes of ω6/ ω3 relation-related ratios of analyzed phospholipid species in SH-SY5Y APPswedish cells compared to mock-transfected cells. Error bars represent the standard error of the mean (SEM). Statistical significance was calculated using two-tailed Student’s *t*-test. (**B**) Fold changes of different phospholipid species containing three double bonds in SH-SY5Y APPswedish cells compared to mock-transfected cells. Error bars represent the standard error of the mean (SEM). Statistical significance was calculated using two-tailed Student’s *t*-test. (**C**,**D**) Fold changes of phosphatidylethanolamines and plasmalogens in respect to their saturation and chain length in SH-SY5Y APPswedish cells compared to mock-transfected cells. Error bars represent the standard error of the mean (SEM). Statistical significance was calculated using two-tailed Student’s *t*-test with * *p* ≤ 0.05 and ** *p* ≤ 0.01.

Interestingly, by further processing the obtained data for mitochondria, we found that the ratio of phosphatidylcholines containing fatty acids with four double bonds (CX:4) to fatty acids containing six double bonds (CX:6) was significantly elevated in the cellular AD model independent of whether the fatty acids of the PC species were linked via an ester- or enolether (PCax CX:4/CX:6). In line, the ratio of PCax CX:4/(CX:5 + CX:6) and PEaa CX:4/CX:6 was significantly elevated in mitochondria of APPswedish transfected cells (Figure 10A). These lipid species include the omega-6 arachidonic acid C20:4 and the omega-3 PUFAs docosahexaenoic acid (DHA, C22:6) and eicosapentaenoic acid (EPA, C20:5) discussed to be beneficial in AD. The observed increase in the ratio of omega-6/omega-3 PUFAs in the cellular AD model might be caused by a reduction in omega-3 PUFAs as we observed a significant reduction in PEaa CX:6 and PEax CX:6 mol% (Figure 10A) for APPswedish expressing cells. The observed reduction in phospholipids containing the omega-3 PUFAs EPA and DHA in APPswedish transfected cells are consistent with reports dealing with DHA in AD-affected individuals. It has been reported that DHA is decreased in AD post mortem brains in brain regions such as hippocampus, white matter, frontal gray matter and pons [83]. Furthermore, it has been shown that AD patients have reduced brain and serum DHA levels compared to age-matched non-demented controls [84,85]. Consistently, fatty acids of phosphatidylethanolamine lipid species with four double bonds were significantly increased in APPswedish cells (Figure 10A). As mentioned above, these lipid species might include aa C20:4. Arachidonic acid was also found to be elevated in APP transgenic mice [86].

An elevated PLA2 activity has been found in brains of AD-affected individuals [49,76,87] and Aβ has been reported to increase PLA2 activity [88,89]. Taken into consideration that an increase in PLA2 activity results in an increase in lyso-phospholipid species, it is not astonishing that we observe elevated lyso-PC and lyso-PE levels in the cellular AD model. However, it also should be mentioned that the impact of lyso-PC in respect to AD is ambiguous in literature because it has also been reported that lyso-PC levels are decreased in brains of AD-affected individuals [40,44,45,46]. Potential contrary observations could be explained by the fact that lyso-phospholipids are, on the one hand, produced by PLA2 activity but are also an important transporter of fatty acids in the brain. An impairment in this transport would result in a decreased lyso-phospholipid level, whereas intracellular, an increased PLA2 activity could result in an increased lyso-phospholipid amount.

Interestingly, lipid species containing three double bonds (CX:3) were also significantly increased in APPswedish transfected cells independent of the phospholipid class bound to (Figure 10B). These lipid species might include the omega-6 FAs gamma-linolenic acid (GLA, C18:3) and dihomo-gamma linolenic acid (DGLA, C20:3) or the omega-3 FA linolenic acid (C18:3). Combined with the data that we found, a reduction in omega-3 PUFAs (see Figure 10A, PEaa CX:6/tot and PEax CX:6/tot), it is more likely that the omega-6 FAs GLA and DGLA are elevated in the cellular AD model. However, it has to be noticed that the sum of the FAs bound to the sn-1 and sn-2 position of the glycerolbackbone is presented and we can therefore not undoubtedly distinguish between single FAs, which is a known caveat of target lipidomics approach by mass spectrometry.

Phosphatidylethanolamine species were significantly increased in mitochondria of APPswedish transfected cells independent of the number of double-bonds, revealing the most prominent effect for PEaa species containing three double-bonds (Figure 10C), which is in line with our finding that lipid species containing three double bonds are significantly increased. Furthermore, PEaa species are consistently significantly elevated independent of the FA saturation level in the cellular AD model and the effect is also independent of the FA chain length (Figure 10C). The results obtained for phosphatidylethanolamine could be also observed for phosphatidylethanolamine plasmalogens. PEae species were consistently elevated in mitochondria of cells expressing the familial swedish mutation, independent of the saturation level, number of double-bonds or chain length (Figure 10D). Most significant effects of the processed data (Figure 10) are observed in phosphatidylethanolamine lipid species, including PEaa and PEae. Interestingly, by comparing APPswedish with APPwt expressing cells, the most prominent differences between the two cell lines exist in phosphatidylethanolamines. These differences might be attributed to changes in APP processing: APPswedish is mainly processed in the amyloidogenic pathway leading to Aβ and transcriptionally active AICD, whereas APPwt is mainly processed in the non-amyloidogenic pathway leading to p3 and AICD, which is discussed to be not transcriptionally active [29,36]. Further APP processing products such as sAPPbeta and/or sAPPalpha or other mechanisms might also contribute to the observed changes in PE species found for APPswedish expressing cells compared to APPwt expressing cells. The increase in phosphatidylcholine, phosphatidylcholine plasmalogens and lyso-phosphatidylcholine in APPwt expressing cells compared to control cells might be also of relevance for Trisomie 21, as the *APP* gene is located on chromosome 21 [90].

In summary, our results underline that lipid changes are specific for organelles such as mitochondria and cannot be easily predicted from analysis contained from complete cells or tissue. In respect to a cellular AD model, e.g., APPswedish transfected neuroblastoma cells, we found an increase in X:3 and also X:4 fatty acids mainly presenting omega-6 fatty acids compared to a decrease in X:5 and X:6 fatty acids consisting of, e.g., the omega-3 fatty acids DHA or EPA. Moreover, at least several lyso-PC and plasmalogens are increased in the mitochondrial membranes, which could be confirmed in another independent AD cellular model overexpressing APPwt. Lipid alterations are accompanied by a change in the function of mitochondria, as alterations in the carnitine carrier system were found. Besides the similarities between the two AD cellular models, differences in the lipid alterations also exist. Most prominently PE revealed the greatest differences between the AD models. Whereas APPswedish transfected cells showed an increase in PE species, PE levels were mostly decreased or showed no alterations in APPwt transfected cells. These changes indicate that in particular, the differences caused by amyloidogenic and non-amyloidogenic processing needs to be investigated in further studies in detail.

## Figures and Tables

**Table 1:** Antibodies used in Western blot analysis.

Label	Epitope	Dilution	2nd Antibody
A5441 (Merck, Darmstadt, Germany)	β-actin	1:5000	anti-mouse IgG HRP (P0260; Dako, Glostrup, Denmark)
ab2900 (Abcam, Berlin, Germany)	EEA1	1:2000	anti-rabbit IgG HRP (W4011; Promega, Walldorf, Germany)
ab25630 (Abcam, Berlin, Germany)	Lamp-1	1:2000	anti-mouse IgG HRP (P0260; Dako, Glostrup, Denmark)
C4721 (Merck, Darmstadt, Germany)	calnexin	1:2000	anti-rabbit IgG HRP (W4011; Promega, Walldorf, Germany)
24127 (proteintech, Manchester, United Kingdom)	SOD2	1:5000	anti-rabbit IgG HRP (W4011; Promega, Walldorf, Germany)
W02	APP/Aβ	5 µg/mL	anti-mouse IgG HRP (P0260; Dako, Glostrup, Denmark)

**Table 2:** Internal standards used for lipidomics analysis.

Name	Vendor
lyso-phosphatidylcholine (19:0 Lyso-PC)	Avanti Polar Lipids (855776P)
phosphatidylcholine (06:0 PC (DHPC))	Avanti Polar Lipids (850305P)
phosphatidylcholine plasmalogens (12:0 Diether PC)	Avanti Polar Lipids (999994P)
phosphatidylethanolamine (08:0 PE)	Avanti Polar Lipids (850699C)
phosphatidylglycerol (17:0–14:1 PG)	Avanti Polar Lipids (LM1204)
octanoyl-L-carnitine d3	Supelco Analytical (53230)
palmitoyl-L-carnitine d3	Supelco Analytical (55107)

## Data Availability

Not applicable.

## Supplementary Materials

The following are available online at https://www.mdpi.com/article/10.3390/biomedicines9081062/s1. Figure S1: Comparison of lipid changes observed in SH-SY5Y APPswedish cells to SH-SY5Y APPwt overexpressing cells. Supplementary Figure S2: Matrix effects. Supplementary Figure S3: Alterations in lipid homeostasis in mitochondrial fractions of SH-SY5Y APPwt cells compared to cells overexpressing APPswedish. Supplementary Figure S4: Alterations in lipid homeostasis in mitochondrial fractions of SH-SY5Y APPswedish cells compared to those in homogenates. Supplementary Figure S5: Comparison of lipid changes observed in SH-SY5Y APPswedish cells to SH-SY5Y APPwt overexpressing cells. Supplementary Figure S6: Comparison of cardiolipin levels in SH-SY5Y APPswedish overexpressing cells to SH-SY5Y mock transfected cells. Supplementary Figure S7: Analysis of phosphatidylglycerol (PG) levels in homogenates of SH-SY5Y APPswedish overexpressing cells in comparison to SH-SY5Y mock transfected cells. Supplementary Table S1: Q1/Q3 masses, declustering potentials (DP) and collision energy (CE) for the analyzed metabolites.
