# Supplementary material for: Targeted Lipidomics of Mitochondria in a Cellular Alzheimer’s Disease Model
Source: Biomedicines. 2021 Aug 21;9(8):1062. doi: 10.3390/biomedicines9081062 (PMC8393816; doi:10.3390/biomedicines9081062)
Supplement: Supplementary file 1 [file biomedicines-09-01062-s001.zip › biomedicines-1300503-supplementary.pdf]

Supplementary Materials

Targeted lipidomics of mitochondria in a cellular Alzheimer´s disease model

Irina Kurokin [1,t], Anna A. Lauer [1,t], Daniel Janitschke [1,t], Jakob Winkler [1], Elena L. Theiss [1], Lea V. Griebsch [1], Sabrina M. Pilz [1], Veronika Matschke [2], Martin van der Laan [3], Heike S. Grimm [1], Tobias Hartmann [4] and Marcus O. W. Grimm [1,4*]

A)

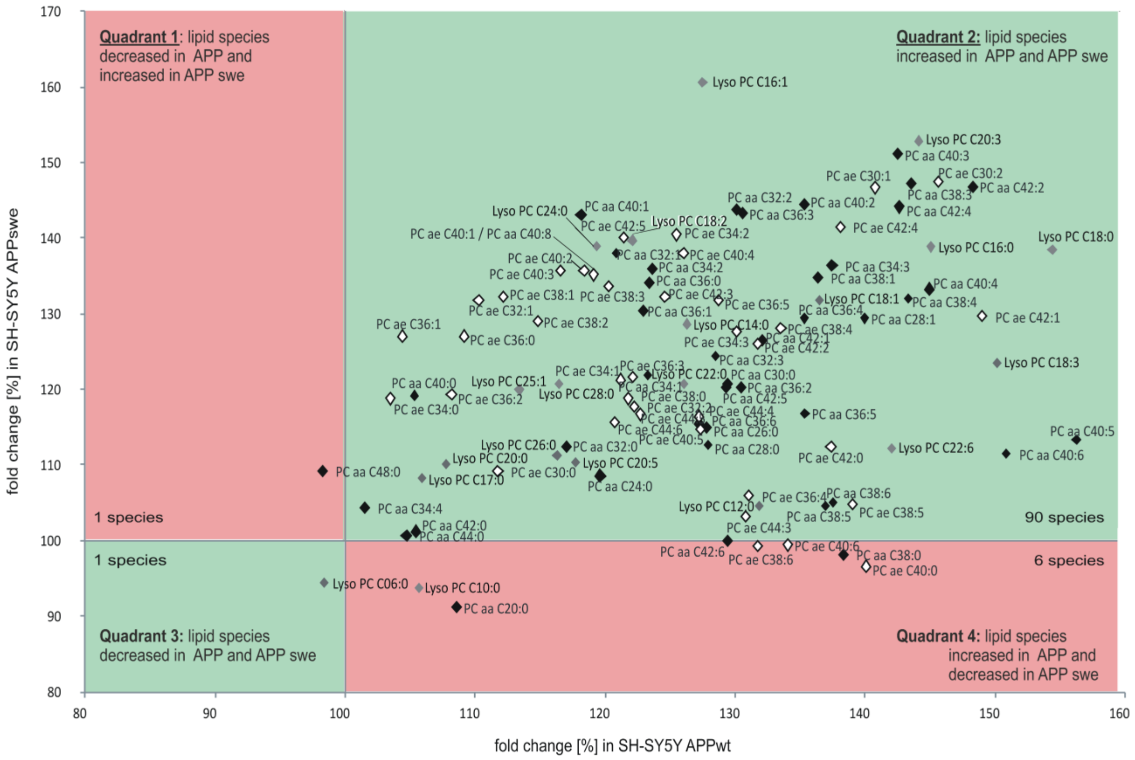

B)

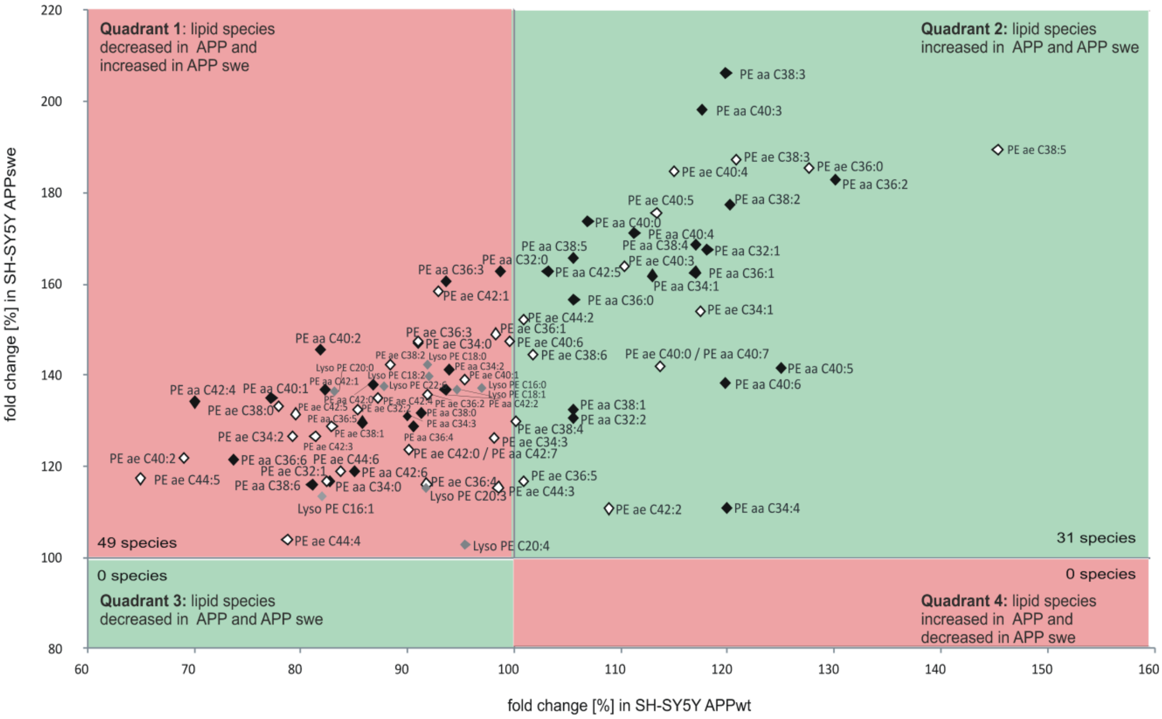

Supplementary Figure S1: Comparison of lipid changes observed in SH-SY5Y APPswedish cells to SH-SY5Y APPwt overexpressing cells. (A) Fold changes of all measured PC species (PCaa, PCae and lyso-PC) in SH-SY5Y APPwt cells (abscissa axis) plotted against fold changes of these species in SH-SY5Y APPswedish cells (ordinate

axis). Quadrants two and three (green) represent PC species that are consistently de- or increased in both cell lines while quadrants one and four (red) include those with different effects in the compared neuroblastoma cell lines. **(B)** Fold changes of all measured PE species (PEaa, PEae and lyso-PE) in SH-SY5Y APPwt cells (abscissa axis) plotted against fold changes of these species in SH-SY5Y APPswedish cells (ordinate axis). Quadrants two and three (green) represent PC species that are consistently de- or increased in both cell lines while quadrants one and four (red) include those with different effects in the compared neuroblastoma cell lines.

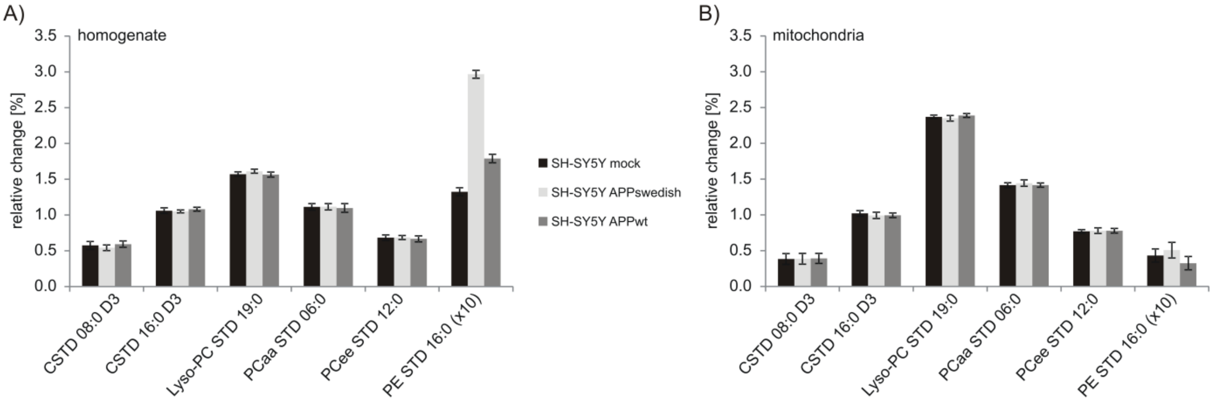

Supplementary Figure S2: Matrix effects. Calculated matrix effects of the measured parameters carnitine, lyso-phosphatidylcholines (lyso-PC), phosphatidylcholines (PCaa), phosphatidylcholines plasmalogens (PCae) and phosphatidylethanolamines (PE) in homogenates (A) and mitochondria (B).

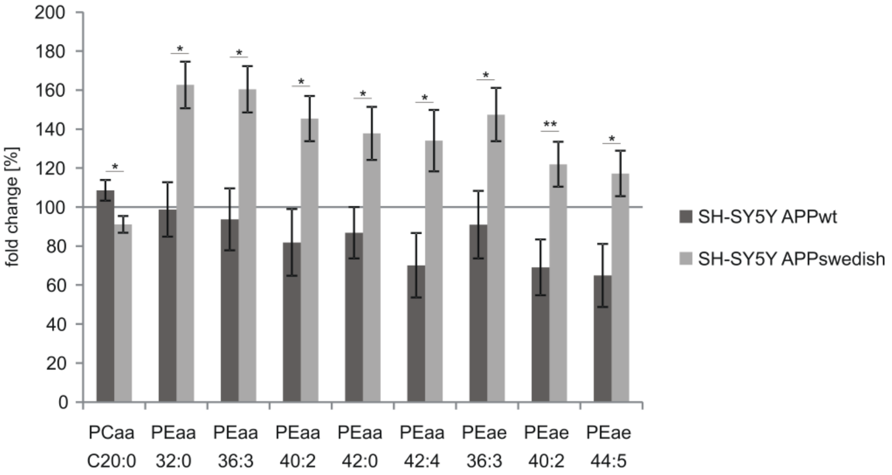

Supplementary Figure S3: Alterations in lipid homeostasis in mitochondrial fractions of SH-SY5Y APPwt cells compared to cells overexpressing APPswedish. Significant differences in PEaa and PEae species are shown as bar charts. Error bars represent the standard error of the mean (SEM). Statistical significance was calculated using two-tailed student´s t-test with * $p \leq 0.05$ and ** $p \leq 0.01$.

A)

	mitochondria				homogenate			
Lipid	PC aa	PC ae	PE aa	PE ae	PC aa	PC ae	PE aa	PE ae
C32:1	137.9	131.9	167.4	116.6	111.3	95.5	106.5	93.8
C32:2	143.8	117.7	130.4	132.5	115.4	95.8	114.3	103.1
C34:1	121.8	121.3	161.8	153.8	103.6	89.0	99.5	99.1
C34:2	135.9	140.5	141.2	126.7	114.4	101.9	117.4	99.2
C34:3	136.4	127.7	131.0	126.3	120.1	99.7	110.0	98.7
C36:0	134.1	127.1	156.6	185.2	107.5	100.7	95.8	112.2
C36:1	130.4	127.1	162.5	149.0	103.6	98.1	96.8	98.0
C36:2	120.3	119.3	182.7	135.6	102.8	91.1	111.1	101.6
C36:3	143.2	121.5	160.5	147.4	121.7	89.4	122.2	96.6
C36:4	129.4	105.9	128.8	116.1	110.3	78.6	94.5	88.1
C36:5	116.9	131.8	129.7	116.7	109.5	99.7	92.6	85.9
C38:0	98.2	118.8	131.7	133.2	76.8	92.6	70.4	84.3
C38:1	134.8	132.2	132.3	128.6	104.7	101.5	103.5	98.3
C38:3	147.2	133.7	206.1	187.3	123.4	96.2	122.7	99.7
C38:4	132.1	128.1	168.7	129.8	117.1	92.3	95.2	92.5
C38:5	104.6	104.7	165.6	189.4	102.3	81.0	89.9	76.2
C38:6	105.1	99.4	116.0	144.6	93.4	78.9	73.9	74.7
C40:0	119.2	96.6	173.6	142.0	90.8	79.7	84.4	72.3
C40:1	143.1	135.2	135.1	139.1	113.7	103.9	109.0	104.1
C40:2	144.4	135.8	145.4	121.9	122.7	107.8	123.3	107.6
C40:3	151.0	135.8	198.1	163.8	128.4	104.8	131.7	107.9
C40:4	133.3	137.9	171.1	184.7	112.6	97.5	95.5	99.0
C40:5	113.2	114.8	141.6	175.7	106.6	82.9	78.2	83.0
C40:6	111.5	99.4	138.2	147.4	98.0	72.8	70.2	70.9
C42:0	101.2	112.4	137.8	123.7	110.1	108.0	71.7	83.8
C42:1	126.5	129.6	137.0	158.3	116.5	112.0	91.8	79.0
C42:2	146.8	126.1	136.8	110.7	123.1	106.3	106.3	84.2
C42:4	144.1	141.5	134.1	134.8	122.1	104.3	95.8	100.3
C42:5	120.2	140.0	162.7	131.5	113.3	96.3	96.3	98.9

B)

	mitochondria		homogenate	
Lipid	Lyso PC	Lyso PE	Lyso PC	Lyso PE
C16:0	138.8	137.2	112.9	114.4
C16:1	160.6	113.5	140.4	135.4
C18:0	138.5	142.4	111.7	87.5
C18:1	131.9	136.8	116.1	125.5
C18:2	139.7	139.8	123.9	125.1
C20:0	110.2	136.5	96.5	109.4
C20:3	152.9	115.2	143.4	159.9
C20:4	122.6	102.8	111.0	125.0
C22:6	112.1	137.4	90.7	97.0

Supplementary Figure S4: Alterations in lipid homeostasis in mitochondrial fractions of SH-SY5Y APPswedish cells compared to those in homogenates. (A) Heat map listing the fold changes of the measured PCaa, PCae, PEaa and PEae species in mitochondria compared to homogenate. The lipid classes are arranged that every line contains the same fatty acids. Therefore effects due to the fatty acid composition can be illustrated more clearly. Green color indicates elevating effects and red color reducing effects. **(B)** Heat map listing the fold changes of the measured lyso-PC and lyso-PE species in mitochondria compared to homogenate. Green color indicates elevating effects and red color reducing effects.

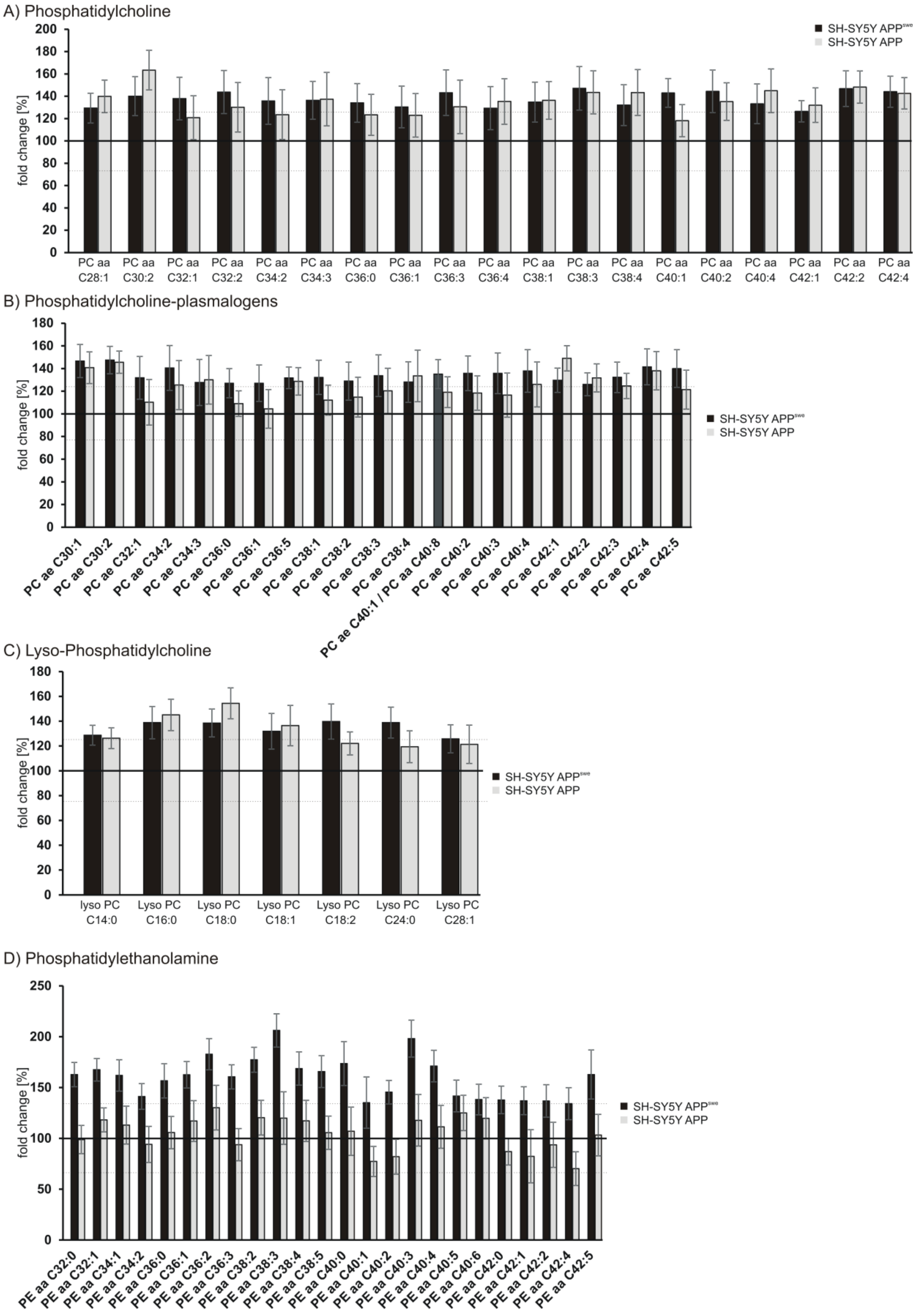
A) Phosphatidylcholine
fold change [%]
SH-SY5Y APP^swe
SH-SY5Y APP
PC aa C28:1
PC aa C30:2
PC aa C32:1
PC aa C32:2
PC aa C34:2
PC aa C34:3
PC aa C36:0
PC aa C36:1
PC aa C36:3
PC aa C36:4
PC aa C38:1
PC aa C38:3
PC aa C38:4
PC aa C40:1
PC aa C40:2
PC aa C40:4
PC aa C42:1
PC aa C42:2
PC aa C42:4
B) Phosphatidylcholine-plasmalogens
fold change [%]
SH-SY5Y APP^swe
SH-SY5Y APP
PC ae C30:1
PC ae C30:2
PC ae C32:1
PC ae C34:2
PC ae C34:3
PC ae C36:0
PC ae C36:1
PC ae C36:5
PC ae C38:1
PC ae C38:2
PC ae C38:3
PC ae C38:4
PC ae C40:1 / PC aa C40:8
PC ae C40:2
PC ae C40:3
PC ae C40:4
PC ae C42:1
PC ae C42:2
PC ae C42:3
PC ae C42:4
PC ae C42:5
C) Lyso-Phosphatidylcholine
fold change [%]
SH-SY5Y APP^swe
SH-SY5Y APP
lyso PC C14:0
Lyso PC C16:0
Lyso PC C18:0
Lyso PC C18:1
Lyso PC C18:2
Lyso PC C24:0
Lyso PC C28:1
D) Phosphatidylethanolamine
fold change [%]
SH-SY5Y APP^swe
SH-SY5Y APP
PE aa C32:0
PE aa C32:1
PE aa C34:1
PE aa C34:2
PE aa C36:0
PE aa C36:1
PE aa C36:2
PE aa C36:3
PE aa C38:2
PE aa C38:3
PE aa C38:4
PE aa C38:5
PE aa C40:0
PE aa C40:1
PE aa C40:2
PE aa C40:3
PE aa C40:4
PE aa C40:5
PE aa C40:6
PE aa C42:0
PE aa C42:1
PE aa C42:2
PE aa C42:4
PE aa C42:5

E) Phosphatidylethanolamine-Plasmalogens

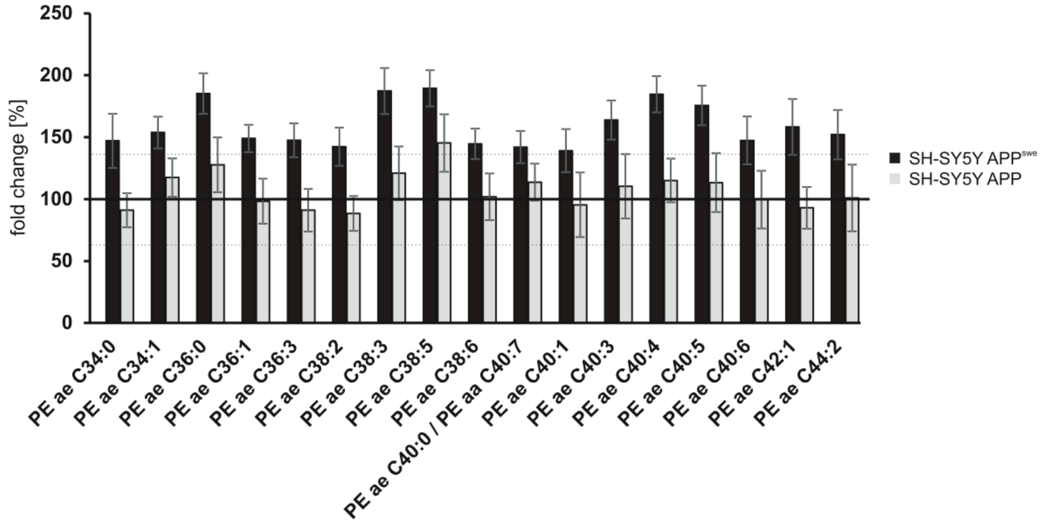

F) Lyso-Phosphatidylethanolamines

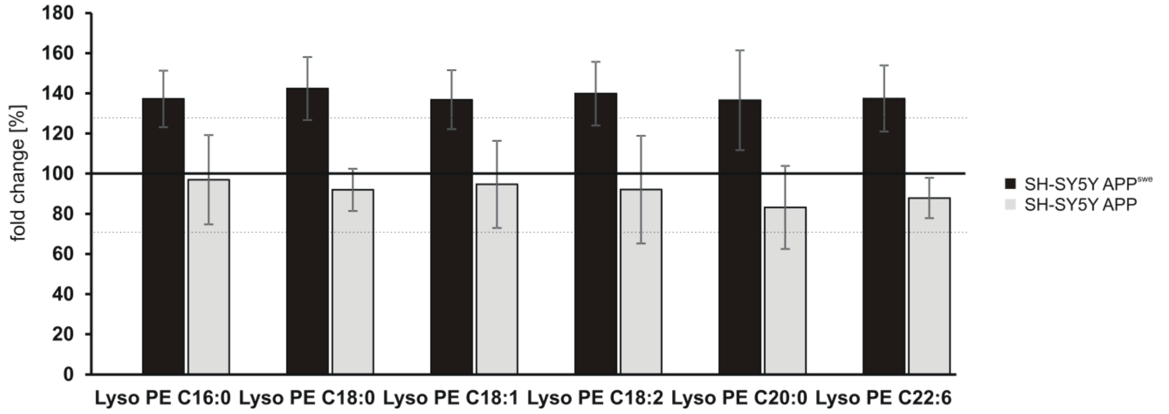

G) Carnitine

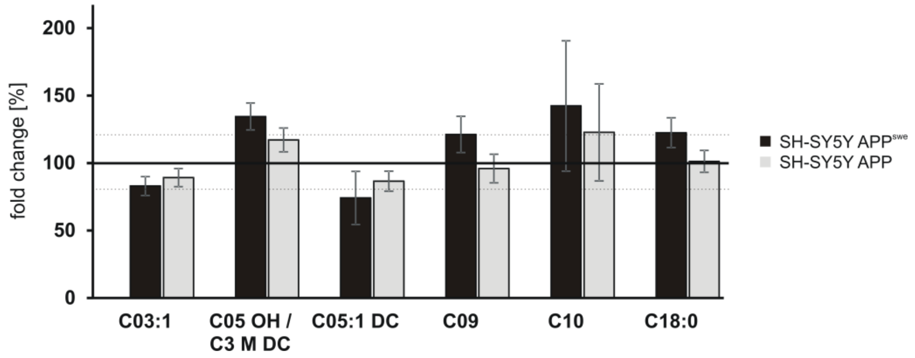

Supplementary Figure S5: Comparison of lipid changes observed in SH-SY5Y APPswedish cells to SH-SY5Y APPwt overexpressing cells. The fold changes of those single phosphatidylcholine **(A)**, phosphatidylcholine-plasmalogens **(B)**, lyso-phosphatidylcholine **(C)**, phosphatidylethanolamine **(D)**, phosphatidylethanolamine-plasmalogens **(E)**, lyso-phosphatidylethanolamine **(F)** and carnitine **(G)** species that are exclusively changed in mitochondria of APPswedish overexpressing cells in comparison to SH-SY5Y APPwt cells. Changes in SH-SY5Y mock cells were set as 100 % and are marked as the corresponding line in the bar chart. Error bars represent the standard error of the mean (SEM). Average SEM was shown as two grey lines in each diagram, respectively. Statistical significance was calculated using two-tailed student´s t-test.

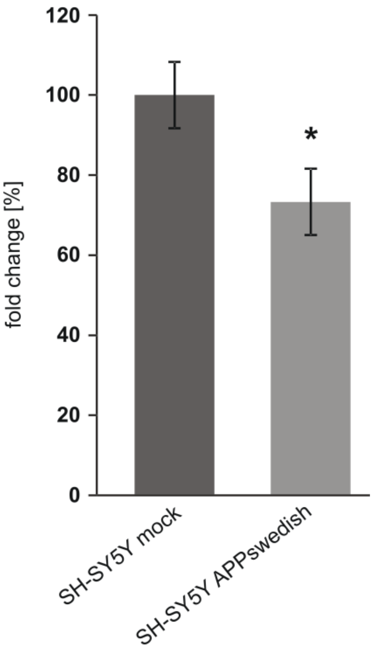

Supplementary Figure S6: Comparison of cardiolipin levels in SH-SY5Y APPswedish overexpressing cells to SH-SY5Y mock transfected cells. The levels of cardiolipin were detected using the cardiolipin assay kit from abcam (ab241036). Changes in SH-SY5Y mock cells were set as 100 %. Error bars represent the standard error of the mean (SEM). Statistical significance was calculated using two-tailed student´s t-test with * $p \leq 0.05$.

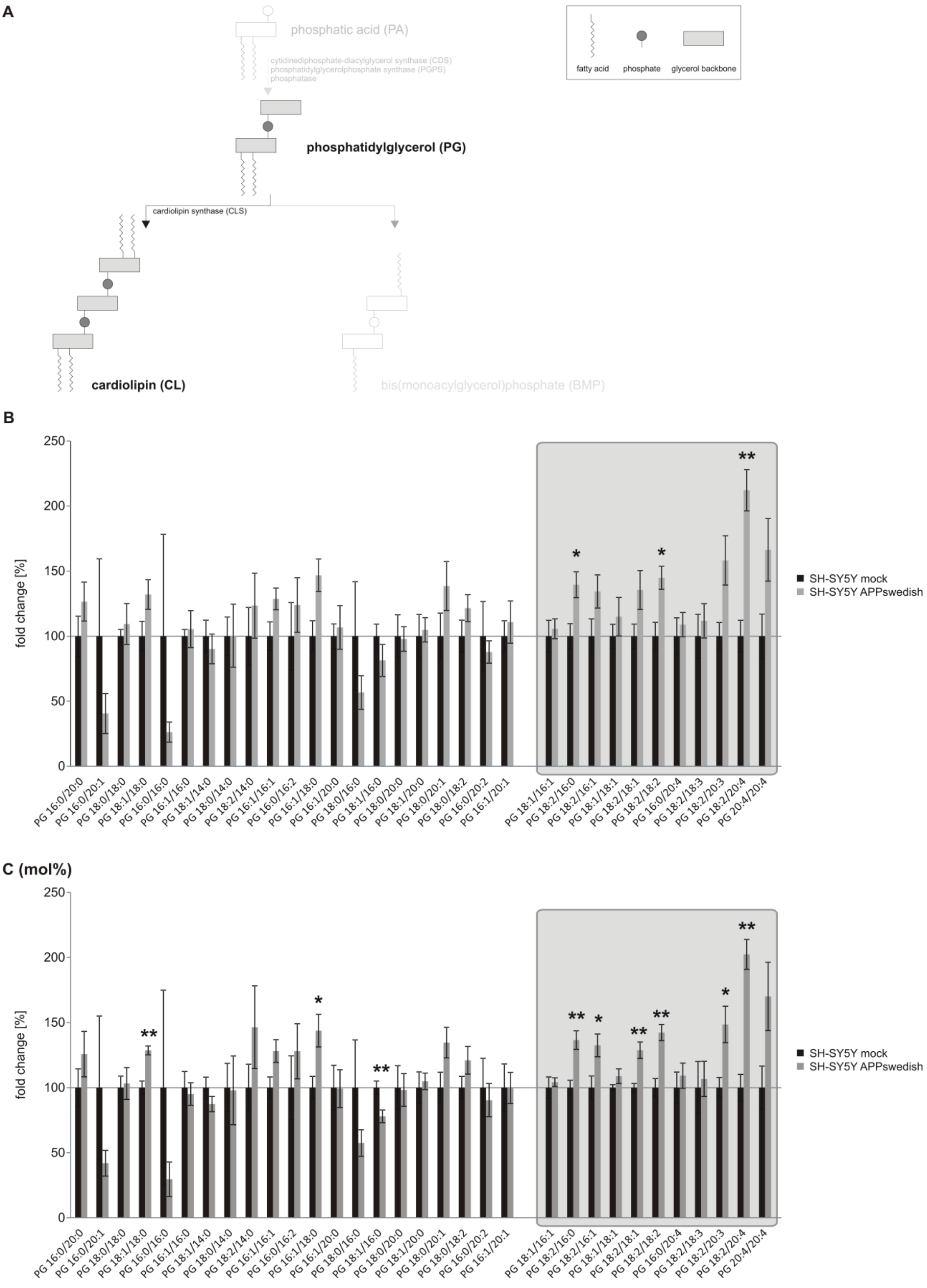

Supplementary Figure S7: Analysis of phosphatidylglycerol (PG) levels in homogenates of SH-SY5Y APPswedish overexpressing cells in comparison to SH-SY5Y mock transfected cells. (A) Schematic overview of the cardiolipin synthesis. The cytidinediphosphate-diacylglycerol synthase (CDS) catalyzes the formation of cytidinediphosphate-diacylglycerol (CDP-DAG) by condensation of CTP and phosphatidic acid (PA). The phosphatidylglycerolphosphate synthase (PGPS) converted CDP-DAG to phosphatidylglycerolphosphate (PGP), which is dephosphorylated by a phosphatase resulting in phosphatidylglycerol (PG). Condensation of one molecule PG and CDP-DAG, respectively, by the cardiolipin synthase leads to the formation of cardiolipin. **(B)** The fold changes of single PG

species in homogenates are shown as bar charts. **(C)** The effects of single PG species independent of PG lipid class effect are presented as bar chart. Changes in SH-SY5Y mock cells were set as 100 %. Error bars represent the standard error of the mean (SEM). Statistical significance was calculated using two-tailed student´s t-test with * $p \leq 0.05$ and ** $p \leq 0.01$.

Supplementary table S1: Q1/Q3 masses, declustering potentials (DP) and collision energy (CE) for the analyzed metabolites.

metabolite	Q1 mass (Da)	Q3 mass (Da)	DP (volts)	CE (volts)
**C0**	162.1	85.1	61	27
**C2**	204.1	85.1	41	27
**C3**	218.1	85.1	46	29
**C03 OH**	234.1	85.1	53	30
**C3:1**	216.1	85.1	49	27
**C4**	232.2	85.1	46	29
**C4 OH**	248.1	85.1	55	32
**C4:1**	230.1	85.1	52	29
**C5**	246.2	85.1	46	29
**C5 M DC**	290.2	85.1	63	37
**C5 OH**	262.2	85.1	58	33
**C5:1**	244.2	85.1	55	31
**C5:1 DC**	274.1	85.1	60	35
**C6**	260.2	85.1	56	27
**C6 OH**	276.2	85.1	61	35
**C6:1**	258.2	85.1	57	33
**C7 DC**	304.2	85.1	66	39
**C8**	288.2	85.1	66	33
**C8:1**	286.2	85.1	63	37
**C9**	302.2	85.1	66	39
**C10**	316.2	85.1	56	37
**C10:1**	314.2	85.1	68	40
**C10:2**	312.2	85.1	67	40
**C12**	344.3	85.1	73	44
**C12 DC**	374.3	85.1	86	45
**C12:1**	342.3	85.1	73	44
**C14**	372.3	85.1	86	45
**C14:1**	370.3	85.1	78	47
**C14:1 OH**	386.3	85.1	81	50
**C14:2**	368.3	85.1	78	47
**C14:2 OH**	384.3	85.1	81	49
**C16**	400.3	85.1	84	51
**C16 OH**	416.3	85.1	87	53
**C16:1**	398.3	85.1	84	51
**C16:1 OH**	414.3	85.1	87	53
**C16:2**	396.3	85.1	83	51
**C16:2 OH**	412.3	85.1	86	53
**C18:0**	428.4	85.1	96	63
**C18:1**	426.4	85.1	89	55
**C18:1 OH**	442.4	85.1	92	57
**C18:2**	424.3	85.1	89	54

**Carnitine Std 1**	291.2	85.1	66	33
**Carnitine Std 2**	403.3	85.1	84	51
**Lyso PC 06:0**	356.2	184	72	31
**Lyso PC 10:0**	412.3	184	76	30
**Lyso PC 12:0**	440.4	184	78	30
**Lyso PC 14:0**	468.3	184	79	30
**Lyso PC 16:0**	496.3	184	81	31
**Lyso PC 16:1**	494.3	184	79	30
**Lyso PC 17:0**	510.3	184	82	31
**Lyso PC 18:0**	524.3	184	85	31
**Lyso PC 18:1**	522.3	184	82	31
**Lyso PC 18:2**	520.3	184	82	31
**Lyso PC 18:3**	518.4	184	82	31
**Lyso PC 20:0**	552.4	184	89	31
**Lyso PC 20:3**	546.3	184	87	31
**Lyso PC 20:4**	544.3	184	85	31
**Lyso PC 20:5**	542.4	184	84	31
**Lyso PC 22:0**	580.5	184	93	32
**Lyso PC 22:6**	568.4	184	90	32
**Lyso PC 24:0**	608.4	184	96	33
**Lyso PC 26:0**	636.5	184	101	34
**Lyso PC 26:1**	634.4	184	98	33
**Lyso PC 28:0**	664.5	184	105	35
**Lyso PC 28:1**	662.5	184	103	35
**Lyso PC Std**	538.6	184	96	40
**PC aa C20:0**	566.2	184	94	31
**PC aa C24:0**	622.4	184	98	33
**PC aa C26:0**	650.5	184	103	35
**PC aa C28:0**	678.5	184	106	35
**PC aa C28:1**	676.5	184	106	35
**PC aa C30:0**	706.5	184	114	37
**PC aa C30:2**	702.5	184	111	37
**PC aa C32:0**	734.6	184	119	39
**PC aa C32:1**	732.6	184	118	39
**PC aa C32:2**	730.5	184	117	38
**PC aa C32:3**	728.5	184	117	38
**PC aa C34:1**	760.6	184	125	41
**PC aa C34:2**	758.6	184	123	40
**PC aa C34:3**	756.6	184	123	40
**PC aa C34:4**	754.5	184	122	40
**PC aa C36:0**	790.6	184	131	43
**PC aa C36:1**	788.6	184	131	43
**PC aa C36:2**	786.6	184	130	42
**PC aa C36:3**	784.6	184	130	42

**PC aa C36:4**	782.6	184	129	42
**PC aa C36:5**	780.6	184	128	42
**PC aa C36:6**	778.5	184	128	42
**PC aa C38:0**	818.7	184	138	45
**PC aa C38:1**	816.7	184	138	45
**PC aa C38:3**	812.6	184	136	44
**PC aa C38:4**	810.6	184	136	44
**PC aa C38:5**	808.6	184	136	44
**PC aa C38:6**	806.6	184	135	44
**PC aa C40:0**	846.7	184	145	47
**PC aa C40:1**	844.7	184	145	47
**PC aa C40:2**	842.7	184	144	47
**PC aa C40:3**	840.7	184	144	47
**PC aa C40:4**	838.6	184	144	47
**PC aa C40:5**	836.6	184	143	47
**PC aa C40:6**	834.6	184	143	46
**PC aa C42:0**	874.7	184	154	50
**PC aa C42:1**	872.7	184	153	50
**PC aa C42:2**	870.7	184	153	50
**PC aa C42:4**	866.7	184	152	50
**PC aa C42:5**	864.7	184	151	49
**PC aa C42:6**	862.6	184	150	49
**PC aa C44:0**	902.7	184	163	50
**PC aa C48:0**	958.8	184	170	50
**PC aa Std**	454.4	184	76	39
**PC ae C30:0**	692.6	184	111	37
**PC ae C30:1**	690.5	184	109	36
**PC ae C30:2**	688.5	184	108	36
**PC ae C32:1**	718.6	184	115	38
**PC ae C32:2**	716.6	184	114	37
**PC ae C34:0**	748.6	184	122	40
**PC ae C34:1**	746.6	184	121	39
**PC ae C34:2**	744.6	184	120	39
**PC ae C34:3**	742.6	184	120	39
**PC ae C36:0**	776.7	184	128	42
**PC ae C36:1**	774.6	184	127	41
**PC ae C36:2**	772.6	184	127	41
**PC ae C36:3**	770.6	184	126	41
**PC ae C36:4**	768.6	184	126	41
**PC ae C36:5**	766.6	184	125	41
**PC ae C38:0**	804.7	184	135	44
**PC ae C38:1**	802.7	184	134	44
**PC ae C38:2**	800.7	184	133	43
**PC ae C38:3**	798.6	184	133	43

**PC ae C38:4**	796.6	184	133	43
**PC ae C38:5**	794.6	184	132	43
**PC ae C38:6**	792.6	184	132	43
**PC ae C40:0**	832.7	184	142	46
**PC ae C40:1**	830.7	184	141	46
**PC ae C40:2**	828.7	184	141	46
**PC ae C40:3**	826.7	184	140	46
**PC ae C40:4**	824.7	184	140	45
**PC ae C40:5**	822.6	184	139	45
**PC ae C40:6**	820.6	184	139	45
**PC ae C42:0**	860.8	184	150	49
**PC ae C42:1**	858.7	184	141	46
**PC ae C42:2**	856.7	184	149	48
**PC ae C42:3**	854.7	184	148	48
**PC ae C42:4**	852.7	184	148	48
**PC ae C42:5**	850.7	184	147	48
**PC ae C44:3**	882.7	184	156	51
**PC ae C44:4**	880.7	184	156	51
**PC ae C44:5**	878.7	184	155	51
**PC ae C44:6**	876.7	184	154	50
**PC ee Std**	594.6	184	74	57
**Lyso PE 16:1**	452.3	311.2	54	23
**Lyso PE 16:0**	454.3	313.2	54	23
**Lyso PE 18:2**	478.3	337.3	54	24
**Lyso PE 18:1**	480.3	339.3	54	24
**Lyso PE 18:0**	482.3	341.3	55	24
**Lyso PE 20:4**	502.4	361.3	55	24
**Lyso PE 20:3**	504.4	363.2	55	25
**Lyso PE 20:0**	510.4	369.4	55	25
**Lyso PE 22:6**	526.4	385.2	56	25
**PE ae 32:2**	674.6	533.5	61	27
**PE ae 32:1**	676.6	535.5	61	27
**PE aa 32:2**	688.5	547.5	64	29
**PE aa 32:1**	690.5	549.5	64	29
**PE aa 32:0**	692.5	551.5	64	29
**PE ae 34:3**	700.6	559.5	64	28
**PE ae 34:2**	702.6	561.5	64	28
**PE ae 34:1**	704.7	563.6	64	28
**PE ae 34:0**	706.6	565.5	64	28
**PE aa 34:4**	712.5	571.5	66	30
**PE aa 34:3**	714.5	573.5	66	30
**PE aa 34:2**	716.5	575.5	66	30
**PE aa 34:1**	718.5	577.5	66	30
**PE aa 34:0**	720.5	579.5	67	30

**PE ae 36:5**	724.7	583.6	66	29
**PE ae 36:4**	726.6	585.5	66	29
**PE ae 36:3**	728.6	587.6	66	29
**PE ae 36:2**	730.6	589.5	67	29
**PE ae 36:1**	732.6	591.5	67	29
**PE ae 36:0**	734.6	593.5	67	29
**PE aa 36:6**	736.6	595.6	68	31
**PE aa 36:5**	738.6	597.6	68	31
**PE aa 36:4**	740.6	599.6	68	31
**PE aa 36:3**	742.6	601.6	68	31
**PE aa 36:2**	744.6	603.6	68	31
**PE aa 36:1**	746.6	605.6	69	31
**PE aa 36:0**	748.6	607.6	69	31
**PE ae 38:6**	750.6	609.6	69	30
**PE ae 38:5**	752.6	611.6	69	30
**PE ae 38:4**	754.6	613.6	69	30
**PE ae 38:3**	756.6	615.6	69	30
**PE ae 38:2**	758.6	617.6	69	30
**PE ae 38:1**	760.6	619.6	70	30
**PE ae 38:0**	762.6	621.6	70	30
**PE aa 38:6**	764.6	623.6	70	31
**PE aa 38:5**	766.6	625.6	70	32
**PE aa 38:4**	768.6	627.6	70	32
**PE aa 38:3**	770.6	629.6	71	32
**PE aa 38:2**	772.6	631.6	71	32
**PE aa 38:1**	774.7	633.6	71	32
**PE aa 38:0**	776.7	635.6	71	32
**PE ae 40:6**	778.6	637.6	72	31
**PE ae 40:5**	780.6	639.6	72	31
**PE ae 40:4**	782.6	641.6	72	31
**PE ae 40:3**	784.6	643.6	72	31
**PE ae 40:2**	786.7	645.6	72	31
**PE ae 40:1**	788.7	647.6	73	31
**PEae 40:0/PEaa 40:7**	790.6	649.6	73	31
**PE aa 40:6**	792.6	651.6	73	32
**PE aa 40:5**	794.6	653.6	73	33
**PE aa 40:4**	796.7	655.6	73	33
**PE aa 40:3**	798.6	657.6	73	33
**PE aa 40:2**	800.7	659.7	73	33
**PE aa 40:1**	802.7	661.7	73	33
**PE aa 40:0**	804.6	663.6	74	33
**PE ae 42:5**	808.7	667.6	75	32
**PE ae 42:4**	810.7	669.6	75	32
**PE ae 42:3**	812.7	671.6	75	32

**PE ae 42:2**	814.7	673.6	75	32
**PE ae 42:1**	816.7	675.6	76	32
**PEae 42:0/PEaa 42:7**	818.7	677.6	76	32
**PE aa 42:6**	820.7	679.6	75	33
**PE aa 42:5**	822.7	681.6	75	34
**PE aa 42:4**	824.7	683.6	75	34
**PE aa 42:2**	828.7	687.6	76	34
**PE aa 42:1**	830.7	689.6	76	34
**PE aa 42:0**	832.7	691.6	76	34
**PE ae 44:6**	834.7	693.6	78	32
**PE ae 44:5**	836.7	695.6	78	33
**PE ae 44:4**	838.7	697.6	78	33
**PE ae 44:3**	840.7	699.6	78	33
**PE ae 44:2**	842.7	701.6	79	33
**Std PE aa 16:0**	468.3	327.2	53	21
**PG 16:0/20:0**	777.6	255.2	-146	-64
**PG 16:0/20:1**	775.7	255.2	-156	-55
**PG 18:0/18:0**	777.6	283.2	-146	-59
**PG 18:1/18:0**	775.7	281.1	-156	-54
**PG 16:0/16:0**	721.6	255.1	-139	-50
**PG 16:1/16:0**	719.6	253.1	-133	-51
**PG 18:1/14:0**	719.6	281.1	-133	-51
**PG 18:0/14:0**	721.6	283.2	-139	-49
**PG 18:2/14:0**	719.6	279.1	-133	-57
**PG 16:1/16:1**	717.6	253.1	-130	-45
**PG 16:0/16:2**	717.6	251.1	-130	-46
**PG 16:1/18:0**	747.6	253.1	-145	-55
**PG 16:1/20:0**	775.7	253.1	-156	-47
**PG 18:0/16:0**	749.6	255.2	-147	-56
**PG 18:1/16:0**	747.6	281.1	-145	-55
**PG 18:2/16:0**	745.6	279.1	-146	-53
**PG 18:1/16:1**	745.6	281.1	-146	-54
**PG 18:0/20:0**	805.6	283.2	-148	-64
**PG 18:1/20:0**	803.6	281.1	-146	-61
**PG 18:0/20:1**	803.6	283.2	-146	-66
**PG 18:1/18:1**	773.6	281.1	-149	-58
**PG 18:0/18:2**	773.6	283.1	-149	-59
**PG 16:0/20:2**	773.6	255.2	-149	-56
**PG 16:1/20:1**	773.6	253.1	-149	-55
**PG Std 17:0/14:1**	705.5	269.2	-160	-54
